# Recent Progress and Trends in the Development of Electrospun and 3D Printed Polymeric-Based Materials to Overcome Antimicrobial Resistance (AMR)

**DOI:** 10.3390/pharmaceutics15071964

**Published:** 2023-07-16

**Authors:** Pablo C. Caracciolo, Gustavo A. Abraham, Ernesto S. Battaglia, Silvestre Bongiovanni Abel

**Affiliations:** Biomedical Polymers Division, Research Institute for Materials Science and Technology (INTEMA), National University of Mar del Plata (UNMdP), National Scientific and Technical Research Council (CONICET), Av. Colón 10850, Mar del Plata 7600, Argentina; pcaracciolo@fi.mdp.edu.ar (P.C.C.); gabraham@fi.mdp.edu.ar (G.A.A.); bat.ernes1998@gmail.com (E.S.B.)

**Keywords:** electrospinning, additive manufacturing, polymeric composites materials, antimicrobial agents, antibiotic-free approaches, antimicrobial resistance

## Abstract

Antimicrobial resistance (AMR) developed by microorganisms is considered one of the most critical public health issues worldwide. This problem is affecting the lives of millions of people and needs to be addressed promptly. Mainly, antibiotics are the substances that contribute to AMR in various strains of bacteria and other microorganisms, leading to infectious diseases that cannot be effectively treated. To avoid the use of antibiotics and similar drugs, several approaches have gained attention in the fields of materials science and engineering as well as pharmaceutics over the past five years. Our focus lies on the design and manufacture of polymeric-based materials capable of incorporating antimicrobial agents excluding the aforementioned substances. In this sense, two of the emerging techniques for materials fabrication, namely, electrospinning and 3D printing, have gained significant attraction. In this article, we provide a summary of the most important findings that contribute to the development of antimicrobial systems using these technologies to incorporate various types of nanomaterials, organic molecules, or natural compounds with the required property. Furthermore, we discuss and consider the challenges that lie ahead in this research field for the coming years.

## 1. Introduction

The significant rise in microbial infections has had a profound impact on global morbidity and mortality rates [1]. Infections are caused by various pathogenic microorganisms, including different strains of bacteria, fungi, parasites, and some viruses [2]. Throughout the 20th century, infections were generally controlled using antimicrobial substances, particularly antibiotics during the so-called “golden era of antibiotics” (1950–1970) when numerous types of these drugs were developed and proven effective [3,4,5]. These conventional therapies involved the administration of low-molecular-weight compounds capable of inhibiting growth and proliferation, mainly by oral or intravenous routes [6]. These substances could be derived from other microorganisms, extracted from plants, or synthesized by chemical processes, offering a broad spectrum of action that made them invaluable in combating infectious diseases [7]. However, the extensive and uncontrolled use of the aforementioned substances has led to the emergence of antimicrobial resistance (AMR) [8]. That is one of the major causes of the resistance, along with inadequate adherence to treatment instructions by most patients and the limited number of new alternative drugs currently being developed to replace ineffective ones [9]. Additionally, issues related to sanitation and the lack of access to clean and potable water significantly contribute to the spread of microbes and the rise of resistance [10].

The AMR phenomenon refers to the ability of bacteria and other microorganisms to evade and resist the effect of drugs. Despite the intrinsic resistance exhibited by microorganisms, there is another form known as “acquired resistance” which occurs when microorganisms possess mechanisms to circumvent the action of these compounds. This can result from enzymatic inactivation, multiple mutations, drug extrusion processes, decreased in the uptake due to changes in the outer membrane permeability, and target modifications, among others [11,12]. It is important to highlight the fact that bacteria, although the primary microorganism associated with the AMR, are not the sole type affected. The phenomenon also impacts viruses, parasites, and fungi. In summary, development of resistance renders antibiotics and other antimicrobial medicines ineffective, making infections increasingly difficult or impossible to treat. This poses a risk of infection spread, leads to associated diseases, and contributes to a significant number of deaths. In this sense, the World Health Organization (WHO) has implemented global programs aimed at designing policies, modifying legislation, and promoting research in the field of AMR to develop novel therapeutic options and treatments. Concerted programs together with other organizations such as the Food and Agriculture Organization of the United Nations (FAO) and the World Organization for Animal Health (OIE) are also underway. Importantly, the WHO annually publishes an updated priority list of pathogens contributing to AMR, serving as a guide for research and development of new antimicrobials, diagnostics, and vaccines [13].

As a promising strategy to avoid the reliance on antibiotics or related substances and to overcome AMR, several researchers are exploring the use of nanomaterial agents with antimicrobial properties for the inactivation of microorganisms. With the emergence of nanotechnology, various types of nanomaterials have been studied and shown antimicrobial activity. Among these, metallic nanoparticles and their derivatives have been extensively developed [14,15]. Other nanomaterials are based on carbon derivatives such as nanotubes, graphene, quantum dots, and organic conducting polymers [16,17,18,19,20]. Additionally, the incorporation of essential oils (EOs) into nanometric systems has been explored, given their well-known activity against several types of microorganisms [21]. From a broader perspective, the possibility to tailor the size, shape, surface charge, and other physicochemical properties of the nanomaterials has contributed to the continuous advancement in this field [22]. More interestingly, in the past five years, there has been a significant focus on incorporating these nanomaterials into polymeric matrices to achieve a controlled stabilization, release, or efficient antimicrobial mechanisms. Electrospinning and additive manufacturing (AM) technologies have emerged as highly explored techniques for the generation of such matrices in the form of micro/nanofiber mats, 3D structures, hydrogels, or their combination [23,24]. Among the advantages of these technologies, it is worth to mention the powerful and great versatility (enabling the incorporation of several nanoparticle kinds to generate composites), the possibility of obtaining materials with high surface area-to-volume ratio, high porosity, and interconnected pores that mimic the extracellular matrix (ECM) for regenerative medicine processes, particularly in damaged and infected tissues [25,26]. In the case of 3D printing as AM technology, precise geometric control at both macro and micro scales, along with the possibility to create new shapes while solvent usage, and saving time, are noteworthy highlights [27,28]. Both techniques have also been explored for the development of antimicrobial textiles and clothing in recent years [29,30,31,32,33]. It is noteworthy that research in this field has significantly increased following the COVID-19 pandemic. Consequently, several protective items and clothes for medical personnel were designed and rapidly developed, with a particular emphasis on using AM techniques [34,35,36,37]. For antimicrobial textiles, it is desirable that they not only effectively combat microorganisms but also possess suitable textile properties. Additionally, they should be safe and durable, allowing for laundering without compromising their quality and antimicrobial effectiveness, among other factors [38].

Herein, we abord the key factors in the generation of advanced electrospun and 3D printed materials that contain substances or nanoparticles capable of replacing antibiotics and similar drugs that contribute to AMR. The aim of the presented strategies is to create antibiotic-free alternative ways of treating infection processes and related diseases by focusing on inactivating different microorganism types. To achieve this, we are especially focusing on, incorporating metallic nanoparticles and derivatives, different kinds of EOs, carbonaceous nanoparticles, and photoactivable molecules, among others, that can serve to reduce the number of microorganism colonies effectively eliminate them. Furthermore, we describe notable advances made by in vivo antimicrobial assays using animal models, taking into account fundamental aspects necessary for the regeneration of damaged tissue due to infection.

## 2. Nanomaterial-Based Antimicrobial Agents

AMR mechanisms are associated with the ability of microorganisms to resist and survive the effects of substances, primarily antibiotics, or to decrease or inactivate the action of antimicrobial agents [39]. The literature predominantly focuses on bacterial mechanisms due to the widespread use of antibiotics. Bacteria possess various mechanisms, including the inactivation of drugs through the action of hydrolytic enzymes (such as β-lactamases, aminoglycoside/fluoroquinolone acetyl transferases, among others), interference with the cell wall, modification of target sites, reduced drug uptake, increased efflux of drugs from microbial cells, and the acquisition of resistance plasmids through transformation or conjugation [40,41,42]. Changes in the diameter or number of porins can also impede antimicrobial entry into bacteria, preventing antibiotics from penetrating the bacterial surface and reaching the cell nucleus. Additionally, the ability of bacteria to form biofilms provides protection against light, dehydration, and drug action, among other factors [43]. In the case of fungi, the mechanisms of antifungal resistance are classified as primary or secondary, and they can be intrinsic or acquired, interfering with drug action at target sites or reducing intracellular drug levels. In particular for *Candida*, one of the most significant resistant fungal pathogens, the induction of efflux pumps that actively remove antifungals from fungal cells is the most common mechanism observed [39,44].

To address the global concerns about AMR, researchers are actively exploring different innovative approaches, including the integration of nanomaterials as antimicrobial agents [45,46,47,48]. Nanomaterials offer a unique combination of physical, chemical, and biological properties that make them promising candidates for combating microbial infections. Moreover, nanofabrication techniques allow the design of a diverse range of nanostructures (i.e., nanodots, nanoparticles, nanosheets, nanocubes, nanodumbells, nanorods, nanowires, nanoshells, nanoneedles, nanocages, nanoflakes, nanoplates, just to mention a few) that exhibit interesting antimicrobial properties. The most common ways to combat the AMR of several pathogens using nanomaterials are related to the cell wall and cytoplasmic membrane disruption, the oxidative stress leaded by reactive oxygen species (ROS) production and also enzymatic inhibition, changes in gene expression and protein deactivation process [49]. This section aims to provide a concise overview of the main antimicrobial agents comprising nanomaterials and shed light on their potential applications in various fields.

In recent years, metallic nanoparticles have gained great attention as potential antimicrobial agents [50]. These nanomaterials exhibit enhanced antimicrobial activities compared to their bulk counterparts, primarily due to their large surface-to-volume ratio and high surface reactivity. With sizes ranging from 1 to 100 nanometers, they possess lower toxicity, excellent stability, and long-lasting action, enabling effective interactions with microorganisms. Silver nanoparticles (Ag NPs) have been extensively investigated for this purpose. However, zinc, copper, gold, titanium, magnesium, and gold nanoparticles have also demonstrated high antimicrobial efficacy against bacteria, fungi, viruses, and other eukaryotic micro-organisms, due to their ability to release metal ions. These ions have toxic effects on microorganisms since they can disrupt various cellular processes, such as enzyme activity, DNA replication, and membrane integrity, ultimately leading to their demise. Additionally, nanoparticles can also interact with microbial cell membranes and induce oxidative stress, causing structural damage and increased permeability, contributing to microbial death. Ag NPs have demonstrated effectiveness against drug-resistant strains, such as methicillin-resistant *Staphylococcus aureus* (MRSA) and vancomycin-resistant *Enterococcus* (VRE). They find applications in wound dressings, medical devices, coatings, and water purification systems. Similar to Ag NPs, copper nanoparticles (Cu NPs) exhibit a broad-spectrum of antimicrobial activity by disrupting the cell membranes, generating ROS, and interfering with intracellular processes. They have shown promising efficacy against bacteria, including *Escherichia coli*, *Pseudomonas aeruginosa*, and *Salmonella* spp. Cu NPs are being explored for various applications, including antimicrobial coatings, air filtration systems, and food packaging materials. Zinc oxide nanoparticles (ZnO NPs) possess inherent antimicrobial properties and biocompatibility. ZnO NPs exert antimicrobial activity through multiple mechanisms, including the generation of ROS, inhibition of microbial enzyme activity, and disruption of DNA synthesis. They have also demonstrated effectiveness against bacteria, fungi, and viruses, including drug-resistant strains such as multidrug-resistant *Acinetobacter baumannii* and *Candida albicans.* Metal oxide nanomaterials, such as titanium dioxide, zinc oxide, and magnesium oxide exhibit antimicrobial properties through their ability to generate ROS upon exposure to light or UV radiation, resulting in photocatalytic activity and antimicrobial effects. Furthermore, metallic nanoparticles can be synthesized using various methods and can be functionalized with antimicrobial agents or surface modifiers to enhance their stability, targeting, and specificity. However, there are some challenges and considerations associated with the use of metallic nanoparticles as antimicrobial agents. One concern is their potential toxicity to human cells. Although they are generally considered safe, extensive research is being conducted to understand their long-term effects and potential risks. Additionally, the stability and controlled release of nanoparticles in biological environments needs to be carefully optimized to ensure their efficacy. Furthermore, the potential development of microbial resistance to nanoparticles should be monitored closely.

Carbon-based nanomaterials, such as graphene, fullerenes, carbon nanotubes (CNTs), and carbon quantum dots (CQDs), have emerged as promising antimicrobial agents in drug delivery, biosensing, and antimicrobial coatings [51,52]. These nanomaterials possess unique physicochemical properties, remarkable stability, and exceptional mechanical strength. Moreover, they exhibit antimicrobial activity by physically damaging microbial cell membranes, disrupting biofilms, and interfering with microbial metabolic processes.

Apart from the aforementioned nanomaterials, various other nanomaterials have shown antimicrobial properties. These include EOs and nanoparticles containing photoactivable molecules. Although EOs are not typically classified as nanomaterials, their constituents can exhibit nanoscale properties and interactions that contribute to their broad-spectrum activity against various microorganisms, including bacteria, fungi, viruses, and protozoa [53]. This antimicrobial efficacy at the molecular level can be attributed to the complex mixture of bioactive compounds that interfere with vital enzymatic processes within the microorganisms, disrupting their metabolic pathways and inhibiting their growth. The small droplet size of EOs emulsions or microemulsions also contributes to their efficient dispersion and interaction with microorganisms, improving their antimicrobial activity. In addition, the encapsulation of EOs within nanoparticles or nanoemulsions improves their stability, controlled release, and targeted delivery.

In the last few years, photoactivable molecules, also known as photosensitizers, have gained interest as potential antimicrobial agents [54]. This approach, known as photodynamic antimicrobial chemotherapy (PACT) or photodynamic therapy (PDT), involves the use of light and photosensitizers to induce a photochemical reaction that generates ROS causing oxidative damage to microbial cells and leading to their destruction. Some common photosensitizers include porphyrins, phthalocyanines, and dyes such as methylene blue and toluidine blue. The non-toxic nature of photosensitizers in the absence of light allows for their safe application. However, there are also some challenges and limitations associated with the use of photoactivable molecules. One limitation is the penetration depth of light, which restricts the application of PACT to superficial infections or those accessible to light. Efforts are being made to overcome this limitation by using light-delivery devices or developing photosensitizers with enhanced light absorption properties.

**Figure 1:**
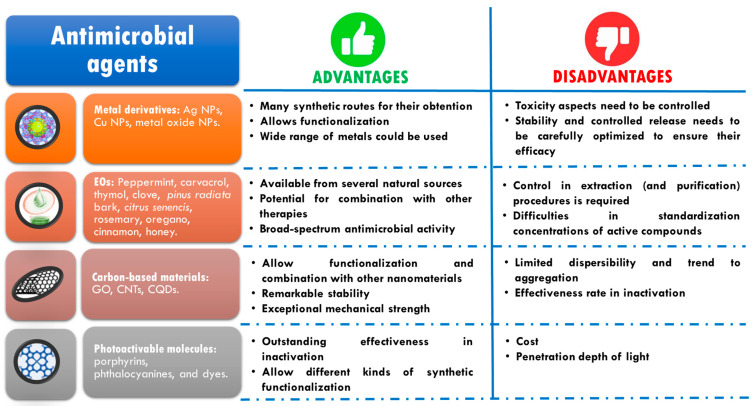
Classification of the most relevant antimicrobial agents used in the last few years and their advantages and critical points.

Figure 1 summarizes the most important antimicrobial agents developed and used in the last few years excluding compounds able to generate AMR. The main advantages and disadvantages of each type are listed.

## 3. Antimicrobial Electrospun Nanofibrous Mats

Electrospinning is a technique based on the electrohydrodynamic process that occurs when a high voltage (typically in the range of 5 to 40 kV) is applied to a polymeric solution.

This high voltage charge leads to the formation of a cone (known as Taylor cone) at the tip of the needle resulting from the electrostatic repulsion between the charged solution and the grounded collector. When the applied voltage surpasses the surface tension of the solution, a thin jet is formed as a result of the balance between the different forces acting on the polymeric drop at the needle tip. The electrostatic instabilities cause the jet to break up into fibers which are subsequently deposited in solid form on the collector forming the electrospun mats as the solvent is evaporated during the process [55]. This versatile, cost-effective, and straightforward technique enables the production of fibrous materials at the nanoscale presenting functional properties that find utility in a wide variety of applications, particularly in the biomedical field. These applications include but are not limited to cell growth, regenerative medicine, and controlled delivery of drugs and bioactive components [56,57,58].

**Figure 2:**
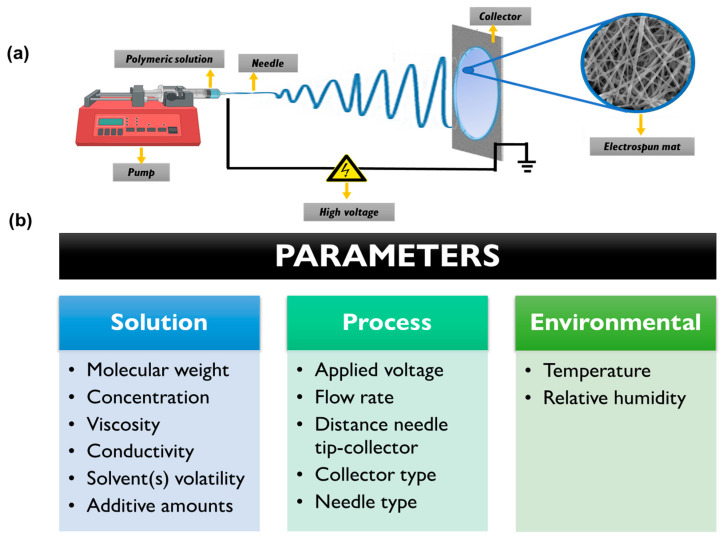
(**a**) Schematic representation of typical electrospinning setup. (**b**) Different parameters which need to be optimized for the obtention of electrospun mats.

The electrospinning technique requires strict control of several key parameters to achieve homogeneous micro/nanofibrous architectures with adequate reproducibility [59]. These parameters encompass various aspects including the properties of the polymeric solution, the electrohydrodynamic process, and the environmental conditions [57,60]. Optimization of solution parameters is crucial and dependent on each specific system, considering factors such as polymer type, inclusion of additives (micro or nanomaterials as well as organic molecules), and solvent composition. Parameters such as concentration and molecular weight, solution conductivity, viscosity, and solvent volatility must be carefully considered [61]. Adjusting and optimizing the flow rate, the distance between the needle tip and collector, and the applied voltage are also important during the electrospinning process [62]. Moreover, the environmental parameters (such as temperature and relative humidity) play a crucial role. Even slight changes in these parameters can impact the collection of the polymeric fibers, morphology, fiber connectivity, and the occurrence of beads or defects in the nanofibrous structure [63]. Figure 2 provides a schematic representation of the electrospinning setup and highlights the parameters that require control and optimization.

The versatility of the electrospinning technique is evidenced in the wide range of materials that can be used (i.e., synthetic and natural polymers, ceramics, metals, and their composites). Furthermore, it allows for the attainment of various fibrous morphologies such as solid, hollow, porous, core-shell, and more. The technique also offers different collector configurations, including flat, cylindrical, rotating, three-dimensional, and their combinations [62,64,65]. This extensive range of possibilities highlights the importance and applicability of the electrospinning technique. The significance of electrospinning is evident in the continuous growth of scientific publications in the field since the beginning of the 21st century [62]. Ongoing developments involve new modifications to the devices and combinations with other techniques, enhancing the capabilities and potential of electrospinning [25,66]. In this section, we focus on the fabrication of sub-micrometer antimicrobial fibrous structures by the electrohydrodynamic technique. We aim to summarize the most significant advances and findings reported in the last five years, highlighting the important contributions made in this area.

### 3.1. Metallic Nanoparticles and Derivatives

As mentioned in the previous section, metallic (and metal oxides and derivatives) nanoparticles present important antimicrobial activity against resistant pathogens. The metallic components can be incorporated or loaded onto polymeric nanofibers by several strategies related to the electrohydrodynamic processes for developing advanced antimicrobial materials [67]. In this sense, some of the most intriguing and recent works found in literature are discussed as follows.

**Figure 3:**
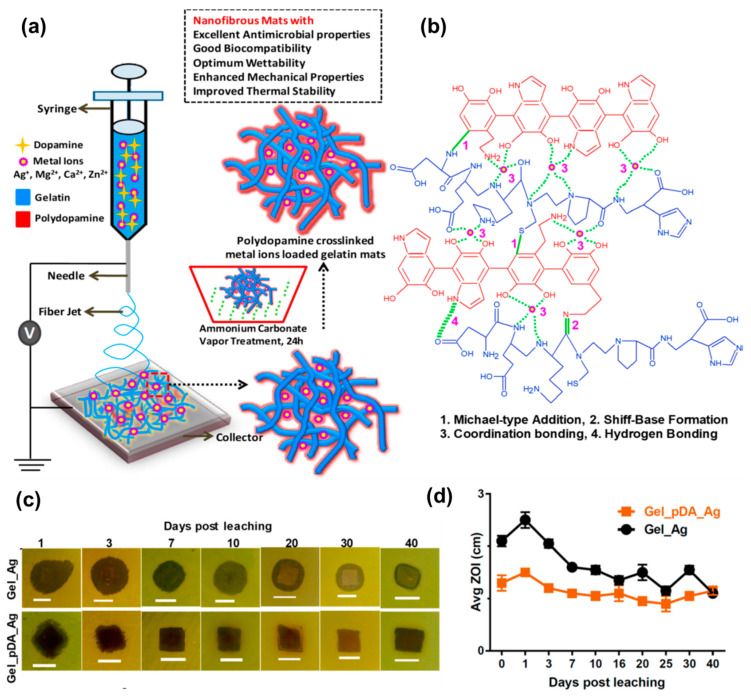
(**a**) Fabrication of PDA cross-linked metal ion loaded GEL mats using electrospinning technique followed by post-spinning ammonium carbonate diffusion. (**b**) Interactions involved in the formation of stable GEL/PDA metal ion architecture for controlled release of ions. (**c**) Representative digital photos showing the long-term antimicrobial activity of GEL-Ag^+^ and GEL/PDA-Ag^+^ mats against *P. Aeruginosa* ATCC 9027 strains. (**d**) Graph showing the changes in ZOI, after soaking in PBS for different time intervals. Note a slight increase in ZOI values for GEL/PDA-Ag^+^ mats immediately after immersion in PBS which later become consistent with increasing time. Each ZOI value represents an average of two independent experiments, determined by disc diffusion assay. Adapted with permission from *ACS Appl. Bio Mater.* **2019**, *2*, 807–823 ([68]). Copyright 2019. American Chemical Society.

By using electrospinning, Leng et al. fabricated polymeric gelatin based (GEL) nanofibrous mats containing dopamine (DA) and different metal ions. A post-treatment step involving exposure to ammonium carbonate vapors was performed to accomplish the crosslinking procedure [68]. The presence of catechol groups in the formed polydopamine (PDA) facilitated the chelation of metal ions (Figure 3a,b). Various materials were obtained by the incorporation of different metal ions, including Ag^+^, Mg^2+^, Zn^2+^_,_ and Ca^2+^.

The authors systematically investigated the processing parameters to achieve the formation of smooth nanofibers. Interestingly, it was found that the wettability of the mats varied depending on the ion incorporated. For instance, silver and magnesium ions increased hydrophilicity, while zinc causes a decrease. The Young’s modulus was highly dependent on the crosslinking degree, while the presence of DA led to an enhancement in Young’s modulus. Regarding the antimicrobial activity of the materials, various assays were conducted against Gram-positive and Gram-negative strains as well as yeast. It was found the GEL/PDA mats did not exhibit any inhibitory effect, indicating a lack of antimicrobial properties when PDA was linked to the natural polymer. Similarly, some of the electrospun mats containing metallic ions did not show any significant antimicrobial activity. However, inhibition zones (ZOI) were detected in the case of materials containing Mg^2+^ against *VRE* and *B. subtilis* strains as well as in the case of zinc and calcium ions against all the Gram-positive strains tested. The GEL/PDA-Ag^+^ mats demonstrated broad-spectrum antimicrobial activity remains against yeast and Gram-negative/positive strains.

To quantify the decrease in colony-forming units (CFU) viability, bactericidal properties were assessed for the aforementioned materials, and positive results were obtained. The antimicrobial properties were retained even after long periods (approximately 40 days), as shown in Figure 3c,d. In a recent study, Mude et al. [69] reported antibiotic-free dressing materials based on poly(ε-caprolactone) (PCL) electrospun mats derivatized with ionic silver using the post-electrohydrodynamic procedure. This was achieved through alkaline hydrolysis of the PCL nanofibrous membranes followed by amine immobilization, and the use of glycidyl trimethylammonium chloride (GTMAC) as a bifunctional linker. Highly dispersed ionic silver was then anchored to quaternary ammonium moieties. The success of the functionalization was corroborated through several techniques including X-ray photoelectron spectroscopy (XPS), small angle X-ray scattering (SAXS), and thermoanalytical techniques, among others. The dressing mats were tested for their anti-infective properties against both Gram-negative and Gram-positive bacteria, demonstrating complete bactericidal action in 90 min for *E. coli* and *S. aureus*, respectively. Near-complete inactivation was achieved in 40 and 60 min, respectively. The in vivo assays were conducted using Balb/c mice model infected with *E. coli*, and promising results were obtained with near-complete healing at day 10 employing the most effective material. Additionally, orderly deposition and arrangement of collagen was observed in the treated wounds. Apart from the antimicrobial characteristics of the materials, it is remarkable to have other multifunctional properties such as excessive biofluid drainage, easy peel-off, porosity, and gas permeation, making them promising candidates for the treatment of chronic wounds and diabetic ulcers.

Metal oxides have also been incorporated into electrospun membranes. One of the most popular metal oxides presenting antimicrobial properties is ZnO. In a study, the loading of ZnO NPs synthesized by a green approach from *Ilex paraguariensis* leaves extracts onto poly(acrylic acid) (PAA) and polyallylamine hydrochloride (PAH) polymeric fibers was achieved [70]. The resulting mats were then annealed (140 °C, 6 h) to crosslink the polymers. On the one hand, the PAA/PAH-ZnO NPs composite fibers presented uniformity and fiber diameters of ca. 230 nm, resembling the ECM structure desired for skin tissue regeneration. On another hand, the morphology and size of ZnO NPs (spherical particles of ca. 18 nm) remained unchanged after the electrohydrodynamic process. The electrospun mats containing the inorganic nanoparticles (0.045 mg of ZnO NPs/0.5 cm^2^ of mat) demonstrated antimicrobial activity, resulting in complete inhibition of *S. aureus* and *E. coli* growth. Notably, the antimicrobial efficacy was significantly higher compared to the control without NPs which exhibited only 40% inhibition against Gram-positive bacteria and 10%inhibition against *E. coli*. Furthermore, Field Emission-Scanning Electron Microscopy (FE-SEM) anlaysis and resazurin assays confirmed the absence of bacteria growth on the fiber surfaces for both bacteria strains. However, one drawback observed during the development of these assays was the agglomeration of some particles in different zones of the fibers, which should be addressed for practical application in wound healing. ZnO NPs were also incorporated onto poly(vinyl alcohol) (PVA) and poly(vinyl pyrrolidone) (PVP) blends before electrospinning [71]. While smooth, beadles, continuous and uniform diameter fibers were obtained for the pristine PVA/PVP fibers, the addition of ZNO NPs at higher concentrations (0.1 g to 1 g of nanoadditives in 9 g of the polymer precursor) resulted in formation of rough and increased size. The ZOI evaluated against *S. aureus*, *E. coli*, *K. pneumonia*, and *P. aeruginosa* bacteria strains showed a concentration-dependent effect of ZnO NPs, with higher inhibition observed against *E. coli* than to the other strains. In another approach, Salmeri et al. generated composites of polyethersulfone/ZnO nanorods mats by growing the nanorods on the surface of the fibers through chemical bath deposition (CBD) [72]. This strategy offers the advantage of direct exposure of bacteria to the oxide, whereas if nanoparticles are inside the fibers, a strictly controlled release is required. The composite mats were tested for their anti-biofilm properties against *S. aureus*, *E. coli*, and *S. epidermidis*. CFU counting revealed better results for *E. coli*. In another research, copper oxide (II) nanoparticles (CuO NPs) with *Momordica charantia* (MC) extract to reduce the toxicity were loaded into PVA electrospun mats. The electrospinning process involved mixing the polymer and MC in water at 10% *w*/*w*, followed by the addition of CuO NPs (ranging from 0 to 0.6% *w*/*w*) after 1 day. Although bead-free homogeneous nanofibers were obtained, the average diameter increased with the amount of CuO NPs, showing a linear relationship with the initial concentrations as confirmed by scanning electron microscopy/energy-dispersive X-ray spectroscopy (SEM-EDS) and transmission electron microscopy (TEM). The antibacterial properties tested by ZOI assays demonstrated better performance against Gram-positive strain (*B. subtilis*) than *E. coli* [73].

**Figure 4:**
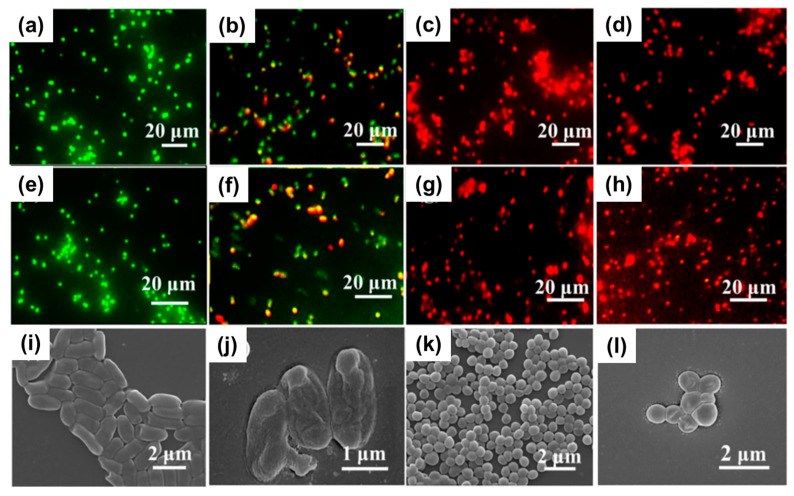
Fluorescent images of (**a**–**d**) *E. coli* and (**e**–**h**) *S. aureus* stained with PLA-Ag_2_[HBTC][im] for (**a**,**e**) 0 h, (**b**,**f**) 2 h, (**c**,**g**) 4 h, and (**d**,**h**) 24 h. FE-SEM images of *E. coli* and *S. aureus* after incubation of 2 h at 37 °C with (**i**,**k**) blank control and (**j**,**l**) PLA-Ag_2_[HBTC][im]. Reproduced with permission from reference *Chem. Eng. J.* **2020**, *390*, 124523 ([76]). Copyright 2020. Elsevier Ltd.

**Figure 5:**
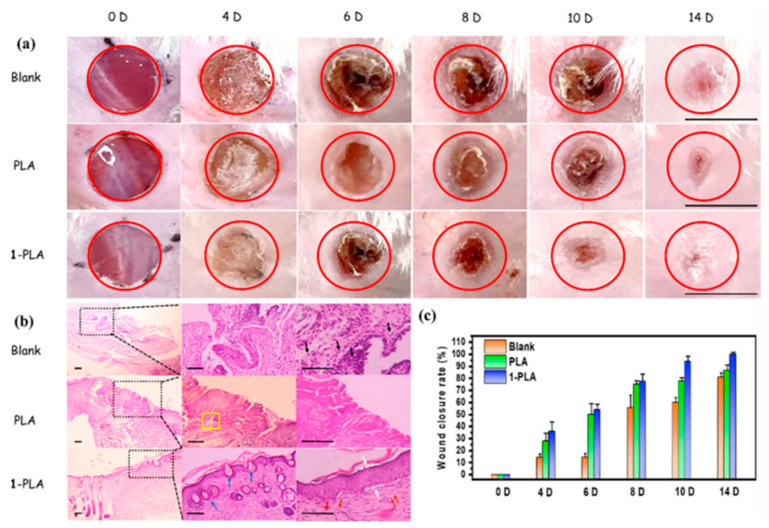
(**a**) Optical photographs of the wound healing process covered with blank, PLA, and PLA- Ag_2_[HBTC][im] (1-PLA). Scale bar: 10 mm. (**b**) H&E staining of skin tissues in wound sites at time points of 14D (Scale bar: 100 μm). Note: Black arrows indicate significant amounts of inflammatory cells; White arrows indicate intact epidermis structure; Blue arrows indicate hair follicles; Red arrows indicate fibroblasts presence; Yellow rectangles refer to disorderly collagen fibers. (**c**) Quantitative analysis of wound closure rates. Reproduced with permission from reference *Chem. Eng. J.* **2020**, *390*, 124523 ([76]). Copyright 2020. Elsevier Ltd.

Metal-organic frameworks (MOFs) enable the linking of metal ions with organic linkers, resulting in structures with important properties such as porosity, biodegradability, and good biological compatibility with living systems. This provides a great opportunity to tune the antimicrobial properties of materials avoiding the use of antibiotics [74,75]. In one study, the incorporation of silver (I) metal-organic (Ag_2_[HBTC][im]) onto poly(lactic acid) (PLA) electrospun mats was achieved [76]. First, the dispersion of the MOFs previously obtained via emulsion methodology was prepared in an organic solvent with the addition of PVP to prevent agglomeration. Then, the dispersed MOFs were carefully added to a PLA solution in the same solvent (dichloromethane) and subjected to an electrohydrodynamic process. The successful fabrication of nanofibers with a diameter of ca. 600 nm was confirmed through EDS, X-ray diffraction (XRD), and Fourier-transform infrared spectroscopy (FTIR). More interestingly, excellent thermal properties added to stability in water medium were observed. *E. coli*, *P. aeruginosa*, *S. aureus*, and *M. smegmatis* strains were used as model bacteria for the analysis of the antimicrobial activity. Remarkable antimicrobial efficiency (>96.5%) was achieved by incorporation of only 1% *w*/*w* of Ag_2_[HBTC][im] within the fibrous structures. The minimum inhibitory concentration (MIC) values for the material were found to be 100–150 mg L^−1^ against *S. aureus*, 50–100 mg L^−1^ against *E. coli*, 0–25 mg L^−1^ against *P. aeruginosa*, and 0–10 mg L^−1^ against *M. smegmatis.* Morphological changes in some of the bacteria strains were detected by FE-SEM examination, revealing the destruction of cell walls upon exposure to the nanofiber mats (Figure 4). Furthermore, the ROS generation, particularly ·O_2_, was confirmed, providing an explanation for the antibacterial mechanism. In vivo assays with infected rats demonstrated the materials’ capability to promote wound healing. After 14 days of treatment, the presence of hair follicles, fibroblasts, and a few inflammatory cells was observed, indicating an accelerated skin tissue regeneration process, as supported by histological analysis (Figure 5). This fact establishes the PLA mats containing Ag_2_[HBTC][im] as potential candidates for broad-spectrum antibiotic-free materials in wound healing applications. Wang and coworkers developed a MOF based on Cu^2+^ (HKUST-1) by solvothermal reaction for their incorporation onto chitosan (CS)/PVA fibers by using a classical electrospinning setup [77]. The resulting fibers presented a diameter of ca. 440 nm with long and continuous morphology as well as smooth surfaces. The successful incorporation of MOFs was confirmed by XRD analysis, which detected the typical diffraction peaks of this material. Furthermore, the presence of new bands in the infrared spectrum when the MOF is present in the CS/PVA blend further confirmed its incorporation. Regarding the antibacterial properties of the materials, the pristine polymeric electrospun mats (without the addition of MOF) showed a kill ratio of ca. 59% against *E. coli* and ca. 67% in the case of exposition to *S. aureus* strain. In vivo assays using male Balb/c rats wound model demonstrated that the HKUST-1-CS/PVA fibers mats could accelerate the wound healing process with minimal inflammation compared to the control group. The studies were conducted over 12 days, with observations recorded on days 6 and 12. The fibrous morphology of the mats plays a crucial role in the healing process by mimicking the ECM. The healing rate for the HKUST-1-CS/PVA fibers (ca. 99.1%) was higher than that the commercial CS dressings and fibers without the MOF. Furthermore, the appearance of the healed wounds was improved in the case of these materials, with no presence of any scar tissue. Examination using hematoxylin and eosin (H&E) staining revealed the presence of fibroblasts and successful re-epithelialization, resulting in the formation of a well-structured epidermal layer after 12 days of treatment.

Although the antimicrobial mechanism of metal nanomaterials and their derivatives is the same, including them in polymeric-based electrospun mats offers several important advantages. For example, these approaches allow for improved biocompatibility since the (nano)agents are incorporated in low proportions compared to the polymer. Additionally, it is favorable for controlling their dispersibility and subsequent release, thereby enhancing the antimicrobial performance and providing biodegradation properties. Finally, the nanofibrous materials, which mimic the ECM, exhibit high porosity, wettability, and permeation properties, making them suitable for use as wound dressings in healing processes [78]. However, it is possible to mention some challenges that need to be addressed in this type of system. These challenges include ensuring the uniform distribution of the agents inside the fibers and, in some cases, preventing the oxidation process that can led inefficacy in the antimicrobial effect.

### 3.2. Essential Oils

One of the most explored approaches to generate antimicrobial materials involves the incorporation of EOs and related components into electrospun mats. Several interesting examples of research using a wide range of EOs are described below.

Unalan et al. obtained PCL fibers using a single nozzle electrospinning setup and loading peppermint essential oil (PEP) at different concentrations (ranging from 1.5 to 6% *v*/*v*) [79]. To avoid toxic solvents, glacial acetic acid was used to dissolve both the polymer pellets and PEP. The resulting fibers had a microscale diameter (1–1.2 μm on average, depending on PEP concentration) and the encapsulation efficiency percentage (EE%) was approximately 40% for all the cases. The study revealed that the addition of the EO did not affect the morphology of the fibers, nor did it lead to aggregation or changes in the wettability. However, the presence of PEP led to a slight increase in the degradation rate during in vitro assays, probably due to a reduction in the intermolecular forces between the polymeric chains by the addition of the EO. In terms of antibacterial activity, the developed materials were found to reduce the viability of *S. aureus* and *E. coli*, with the antimicrobial activity increasing with higher PEP concentration. While both bacteria strains being inhibited, the effect was more pronounced against the Gram-positive strain. This could be attributed due to the reduced penetration of the antibacterial compounds in *E. coli* due to its membrane nature (lipopolysaccharide layer).

PCL was also used to load other EOs such as carvacrol (CAR) and thymol (THY) [80]. For example, the polymer and each EO (ca. 20% *w*/*w*) were dissolved in separate organic solvents mixtures to obtain nanofibers with a considerably reduced diameter compared to the reported by Unalan et al. [79]. The drug loading determined by Gas Chromatography (GC) and by Proton Nuclear Magnetic Resonance (^1^H-NMR), reached a value of ca. 16–17% in both cases. However, when calculating the EE%, PCL-THY presented a higher value compared to PCL-CAR (85% versus 77%). The release of the active component after 24 h was in the range of 5–7%. The antimicrobial activity for both materials was tested versus pathogenic bacteria to determine the MIC and the minimum bactericidal concentration (MBC). In this sense, the obtained results indicated that the release doses of THY from the electrospun mats were below the MIC values required to inhibit bacterial growth. Similarly, nanofibrous materials based on PCL loaded with THY were reported by García-Salinas et al. some years ago [81]. THY-loaded PCL nanofibers displayed an average diameter of ca. 300 nm, achieving a total porosity (pore volume/total volume) close to 73%. Additionally, the porosimetry studies revealed pore sizes in the order of 1.9 µm, which were generated by the random orientation of the polymeric fibers. The EO was loaded in a ca. 15% presenting an EE% of more than 70%. Kinetic experiments demonstrated a burst release of THY in the first few minutes and data analysis using different models led the authors to conclude that the Peppas and Shalin model provided the best fit (R^2^ correlation coefficient = 0.945). The novelty of the research lay in the testing of the in vivo bactericidal capacity of the electrospun mats using old male SKH1 hairless mice infected with *S. aureus* (ca. 10^7^ colonies). The study compared the performance of the THY-loaded PCL mats with pristine PCL mats, free THY, and chlorhexidine (CLXD, a model antiseptic). The results emphasized the importance of the THY encapsulation and the need for contact between bacteria and the electrospun mat to achieve superior antimicrobial action compared to the other conditions. Furthermore, histopathological and immunohistochemical results demonstrated that wounds treated with pristine PCL exhibited diffuse necrotizing dermatitis characterized by massive infiltrations of inflammatory, severe tissue necrosis and the growth of coccoid bacteria. In contrast, when THY-loaded PCL mats were used, non-inflammatory success and only a few layers of coagulative necrosis (less than in the case of CLXD treatment) were observed.

**Figure 6:**
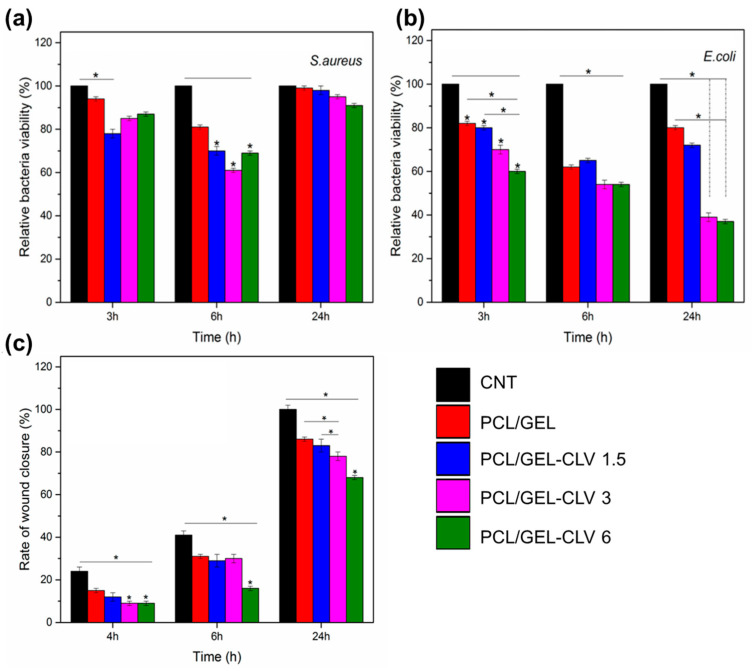
Antibacterial activity of PCL/GEL and CLV-loaded PCL/GEL nanofiber mats after 3, 6, and 24 h incubation with (**a**) *S. aureus* (Gram-positive) and (**b**) *E. coli* (Gram-negative) bacteria (*n* = 3, samples in triplicate, * *p* < 0.05). (**c**) In vitro wound healing assay in the presence of CONTROL, PCL/GEL, PCL/GEL-CLV 1.5, PCL/GEL-CLV 3, and PCL/GEL-CLV 6 in 24 h (*n* = 3, samples in triplicate, * *p* < 0.05). Note: 1.5, 3, and 6 represent % *v*/*v* of CLV respect to PCL. Reprinted with permission from *Pharmaceutics* **2019**, *11*, 570 ([82]). Copyright 2019. MDPI.

PCL combined with GEL is another material reported for the incorporation of EOs. For instance, Boccaccini and coworkers loaded clove essential oil (CLV) into these polymeric fibers [82]. The resulting materials had smooth and relatively thin nanofibers (ca. 250–300 nm of diameter) and the EE% for CLV was more than 50% for all the tested concentrations. Although the antimicrobial effect was assayed against both Gram-negative and Gram-positive strains, the viability reduction in bacteria was significantly more pronounced in the case of Gram-negative strain (*E. coli*) (Figure 6a,b). However, in vitro wound healing assays revealed that an increase in CLV concentration inhibited the migration and subsequent proliferation of normal human dermal fibroblast (NHDF) in a dose-dependent manner, but all the samples were able to close the wound (Figure 6c). Additionally, in materials based on PCL and GEL, the incorporation of *Pinus radiata* bark extracts (PEs) at two different concentrations did not yield satisfactory results, with inhibition percentages of less than 30% observed against *E. coli* and *S. aureus* [83]. This could be attributed to the low concentrations of PE in the nanofibers (less than 0.5% *w*/*w*) combined with the fast release of the active compound when the materials were exposed to the simulated medium. Abdollahi et al. reported an approach for the impregnation of nanogels containing EOs onto PCL electrospun nanofibers [84]. *Citrus sinencis* essential oil (CSEO) was chosen as the antimicrobial agent. Gas-chromatography-mass spectroscopy (CG-MS) analysis revealed that limonene was the major component (ca. 62%). Other detected ingredients present in the range of 3–5% included trans-p-2,8-menthadien1-ol limonene oxide, trans-limonene oxide, cis-, and trans-carveol. For the synthesis of nanoemulsions, several compositions were tested using Span, Tween, ethanol, and water, and a formulation with a particle size of ca. 125 nm was selected. After the impregnation of the nanogels onto the surface of PCL nanofibers, the antimicrobial activity was tested, demonstrating excellent effectiveness in inhibiting *P. aeruginosa*, *E. coli*, *K. pneumonia*, and *S. aureus* strains.

Rosemary and oregano oils are other types of EOs that have been incorporated into cellulose acetate (CA) micro/nanofibers at several concentrations (ca. 1–5% *v*/*v*) using acetone as a solvent by electrohydrodynamic processing [85]. The grafting of the EOs onto the polymeric structure of the fibers was corroborated by Raman spectroscopy. The antimicrobial activity of the electrospun mats containing both types of EOs was assayed against *S. aureus*, *E. coli*, and *C. albicans*, finding that the performance regarding the antibacterial activity was dependent on the EO concentration. The authors demonstrated that the antibacterial activity was dependent on the concentration of EOs. Furthermore, they found oregano oil exhibited higher antimicrobial activity compared to rosemary oil, and both EOs were more effective in reducing the viability of *C. Albicans* compared to the bacteria strains. Pruthi and coworkers conducted research to inhibit the growth of *C. glabrata* and *C. albicans* by incorporating cinnamaldehyde, derived from cinnamon essential oil, into gellan/PVA nanofibers [86]. The nanofibers exhibited an encapsulation efficiency of approximately 17%, and the release of cinnamaldehyde was completed within the first hour. The authors explained that this burst release is desirable for antimicrobial components as it allows for swift inhibition of microbial growth. The results demonstrated that the nanofibers were effective in eradicating approximately 90% of *C. glabrata* and over 50% of *C. albicans*. Moreover, the materials also showed efficacy against *S. aureus* and *P. aeruginosa* bacteria strains. Recently, EOs from *Ocinum basilicum* and *Ocinum gratissinum* were successfully encapsulated into PLA nanofibers by blow spinning method [87]. By mixing different proportions of the mentioned EOs, it allowed the bioactive materials to display effectiveness as an antifungal against *Aspergillus ochraceus* and *Aspergillus westerdjikiae*. According to the literature, EOs have demonstrated the ability to inhibit and control the main virulence factors associated with pathogenic fungal species, particularly *Candida*. These factors include the formation and proliferation of hyphae, as well as the eradication of mature biofilms, which are responsible for the production of harmful enzymes such as hydrolytic proteases, phospholipases, and hemolysins [88]. In this context, one alternative approach is the utilization of nanomaterials for the specific-targeted delivery of antifungal agents that are not prone to causing antimicrobial resistance. This approach aims to facilitate the inactivation or death of fungal cells through interference with the integrity of cell membranes, promotion of ROS, and alteration of membrane permeability [89].

**Figure 7:**
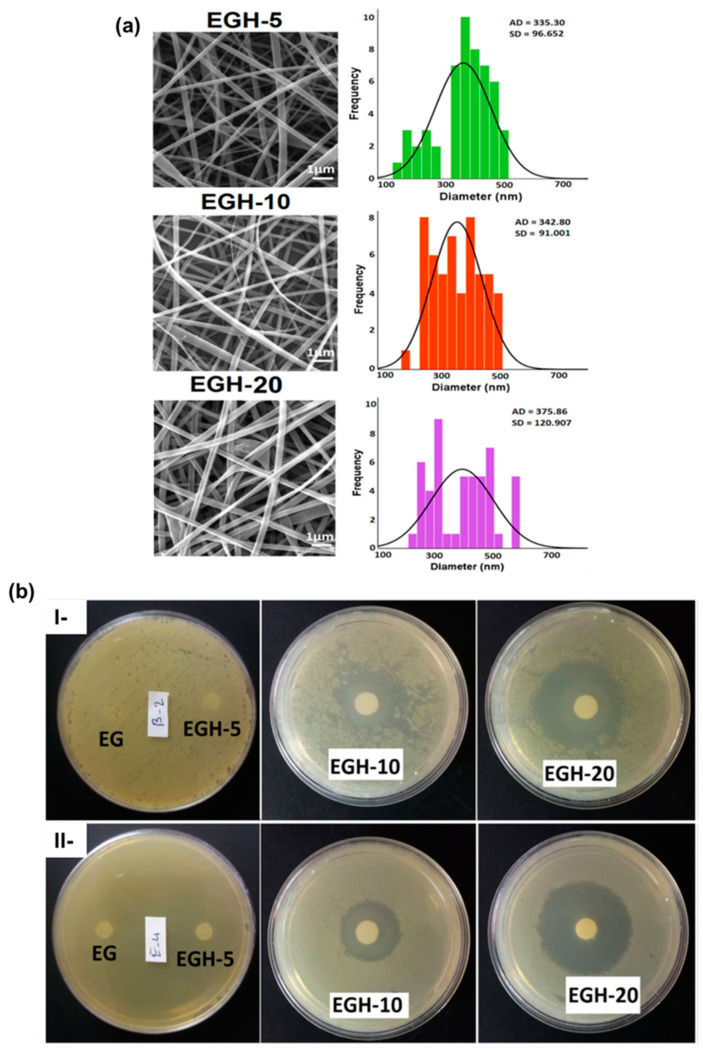
(**a**) SEM images and average diameter and distributions of nanofibers with various content of honey: 5% (EGH-5), 10% (EGH-10), and 20% (EGH-20) (Scale bar: 1 μm). (**b**) The antibacterial activity of blank nanofibers and nanofibers with different content of honey 0% *w*/*w*% (EG), 5% *w*/*w* (EGH-5), 10% *w*/*w* (EGH-10), and 20% *w*/*w* (EGH-20) against (**I**) *S. aureus* and (**II**) *E. coli* bacteria. Note: EC: ethyl cellulose; GT: gum tragacanth; EGH: loaded honey EC/GT nanofibers. Adapted with permission from reference *Colloids Surf. A: Physicochem. Eng. Asp.* **2021**, *621*, 126615. ([91]). Copyright 2020. Elsevier Ltd.

Other authors reported a combinational method to produce nanofibers using hyaluronic acid (HAc)/PVA/poly(ethylene oxide) (PEO) along with the incorporation of cinnamon essential oil (CEO) and metallic nanoparticles (ZnO NPs) [90]. The chosen setup for fiber formation was emulsion electrospinning, which allowed the obtention of nanometric uniform fibers. The incorporation of antimicrobial compounds was confirmed using spectroscopic techniques (FTIR) and XRD. On the one hand, the in vitro inhibition and bactericidal effect against *S. aureus* were observed due to the action of the metallic nanoparticles and the controlled release of CEO. The bacterial count reduction ranged from 90–99.999% depending on the time of evaluation (ranging from 3 to 24 h). On the other hand, the in vivo performance against the same strain in an early stage of wound healing was evaluated using a combination of morphological, microbiological, and histological assays in rats’ (Wistar albino) full-thickness dorsal excision wounds. Despite all the tested materials showing the inhibition of bacteria at day 11, the formulation that combined the ZnO with CEO revealed a greater reduction at the early stage (day 3) compared to the nanofibrous mats containing only metallic particles or EO. The synergistic combination of both antimicrobial agents showed a more pronounced epithelialization process, including the formation of the thick epidermal layer, blood vessels, hair follicles, and sebaceous glands as well as deposition of collagen fibers. This effect could be attributed to the generation of ROS, with exerted antimicrobial activity and the stimulation of angiogenic growth factors expression due to the presence of nanoparticles, in addition to the antimicrobial, antioxidant, and anti-inflammatory properties of CEO. Recently, the loading of honey, which has antioxidant presenting antibacterial properties, onto ethylcellulose (EC)/gum tragacanth (GT) nanofibers was reported [91]. Honey concentrations ranging from 5 to 20% *w*/*w* were incorporated using a green solvent (ethanol). Fibers presenting diameters of less than 350 nm with homogeneous and bead-free structures were successfully obtained by single nozzle electrospinning using high flow rates (ca. 5 mL h^−1^) as demonstrated in Figure 7a. Improvements in the thermal properties by the addition of honey to EC/GT polymers were observed, probably due to the interactions between the natural component and the nanofibrous structure by hydrogen bonding as revealed by FTIR spectroscopy. However, increasing honey concentrations resulted in reduced swelling behavior, increased hydrophobicity, and longer degradation times of the polymeric mats. Despite these effects, the materials exhibited improved mechanical strength due to interactions between interpolymer bonds. The antimicrobial activity against *S. aureus* and *E. coli* bacteria was found to be dependent on the honey content, as shown in Figure 7b. Moreover, the EC/GT honey-loaded nanofibrous materials demonstrated enhanced antioxidant activity, as well as improved cell attachment and proliferation of fibroblasts, making them potential candidates for the regeneration of infected tissues.

### 3.3. Carbon-Based Nanomaterials

Carbonaceous components have emerged as potential candidates for the development of antimicrobial nanofibrous mats. Among other advantages, several carbon-based structures have shown remarkable antibacterial and antifungal properties that added to the versatility of the synthetic approaches [17].

**Figure 8:**
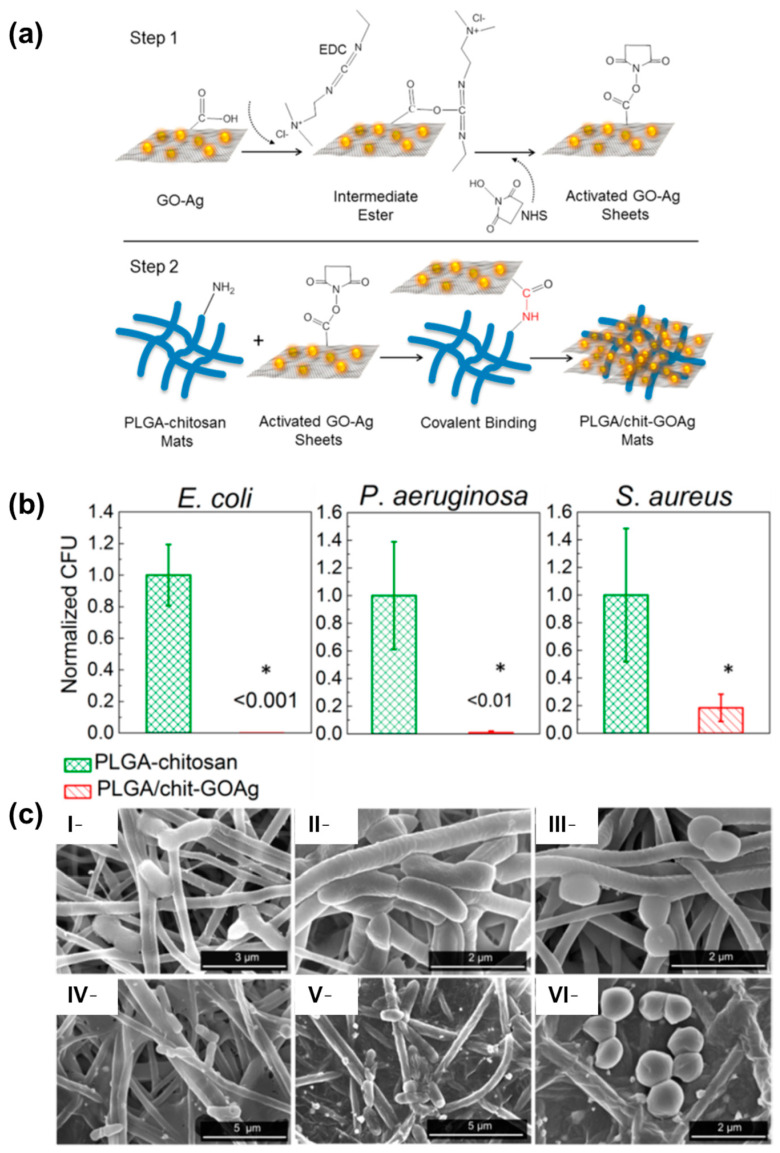
(**a**) Schematic diagram showing the protocol for covalent binding of GOAg nanocomposite to the PLGA/CS nanofibers surface. Step 1: the native carboxylic groups on GO sheets react with EDC and NHS to form an intermediate-activated ester at pH 5.0 (MES buffer). Step 2: the native free amine groups present in the PLGA/CS nanofibers react with the intermediate ester, leading to the formation of a stable amide bond that covalently links the GO-based materials with the surface of the electrospun mats. (**b**) Number of attached viable bacteria cells on PLGA/CS (control) and PLGA/CS-GOAg after exposure to Gram-negative (*E. coli* and *P. aeruginosa*) and Gram-positive (*S. aureus*) bacteria. The values were normalized by dividing the number of live attached cells on PLGA/CS-GOAg sample by the number of attached cells on the nonmodified PLGA/CS (control). The statistical significance of the inactivation rates was confirmed through a hypothesis *t*-test. All the inactivation rates of PLGA/CS−GOAg were significantly different from the control PLGA/CS with *p*-values of less than 0.05, indicated by *. (**c**) SEM micrographs displaying the morphological characteristics of *E. coli* (**I**,**IV**), *P. aeruginosa* (**II**,**V**), and *S. aureus* (**III**,**VI**) cells after contact with PLGA/CS (**I**–**III**) and PLGA/CS-GOAg (**IV**–**VI**) modified nanofibers. Adapted with permission from reference *ACS Appl. Mater. Interfaces* **2015**, *7*, 12751–12759 ([98]). Copyright 2015. American Chemical Society.

The use of graphene oxide (GO) as an additive in the PVA/CS electrospun mats was reported by Tamayo Marín et al. [92]. GO was obtained by reaction of graphite in a concentrated sulfuric acid medium with addition of KMnO_4_ for three days, followed by centrifugation as was previously described by Tovar and coworkers [93]. In a green solvent approach, CS was dissolved in an aqueous acetic acid solution and PVA was dissolved in water, with the dispersion of GO in low concentration (ca. 0.5–1% *w*/*w* respect to CS). The electrohydrodynamic process resulted in the formation of mats with randomly oriented uniform nanofibers in the range of 150–200 nm in diameter and exhibiting an interconnected porous structure within the mat. Interestingly, the increase in GO content led to a higher material stability in simulated body fluid (SBF), revealing the hydroxyapatite formation on the surface of the material after 14 days. While blank controls (without GO) and matrices containing GO concentrations of 0.5%, did not exhibit any antibacterial effect, the pathogens were effectively inhibited when the concentration of GO reached 1%. In this sense, *B. cereus*, *S. aureus*, *Salmonella* spp., and *E. coli* were all inactivated when the mentioned concentration of carbonaceous material was incorporated onto PVA/CS electrospun mats, owing to the destabilization of the cell membranes. This behavior could be attributed due to the promotion of lipid peroxidation induced by the natural oxidative nature of GO, which is concentration-dependent. Additionally, when the materials were implanted in an animal model (Wistar rats), the healing process occurred normally, with evidence of tissue architecture recovery. However, it is worth noting that the use of scaffolds containing 0.5% and 1% of GO resulted in the detection of inflammation. Considering this point, further studies are required to ascertain the feasibility of these developed materials and elucidate their application in subdermal locations or for topical use on infected skin. In another work, GO was decorated with silver nanoparticles and further employed for surface modification of electrospun mats based on poly[lactide-co-glycolide] (PLGA)/CS, taking into consideration previous reports that showed strong antibacterial activity against Gram-negative and Gram-positive bacteria strains. The authors modified the classical Hummers method to obtain GO and then synthesized the Ag NPs by reducing a silver nitrate precursor with NaBH_4_ using sonication to achieve an effective contact between silver ions and oxygenated functional moieties at the surface of the carbonaceous material. The Ag NPs formed on the GO sheets (size of ca. 7 nm) were then used to functionalize PLGA and PLGA/CS blend electrospun mats with a nanofibrous structure (diameters of around 300 and 800 nm, respectively). The surface modification was made with GO and GOAg by a two-step process (refer to Figure 8a). First, the GO-based materials were activated by exposing them to N-ethyl-N′-(3-(dimethylamine)propyl) carbodiimide/N-hydroxysuccinimide (EDC/NHS), which converts the -COOH moieties into an intermediate reactive ester. Subsequently, the activated GO and GOAg sheet suspensions reacted with primary amine groups present on the fibers´ surface to form amide bonds. The obtained materials underwent microscopic and spectroscopic characterization confirming the homogeneous distribution of the particles on the fibers as well as the success in the chemical modification (determined by Raman analysis). The PLGA/CS-GOAg mats were used to assay the antibacterial effectiveness against *S. aureus*, *E. coli*, and *P. aeruginosa*, comparing with the performance of pristine PLGA/CS electrospun mats (Figure 8b). It was found that the inactivation resulted in higher Gram-negative strains (over 98%) than for *S. aureus* (ca. 79.4%). This is probably due to the mentioned differences in the structural morphologic aspects of the bacterial cell wall, the Gram-positive strains being more protected by the thicker peptidoglycan layer composition. Furthermore, the electronic microscopy analysis (Figure 8c) demonstrated the irreversible damage of the cells upon contact with the modified electrospun mats. The authors proposed a mechanism based on the surface oxidation of the nanoparticles, leading to the generation of Ag^+^ ions and subsequent production of ROS upon direct contact, which destabilizes the bacterial cell membranes. However, the key role of GO lies in its large surface area, which enhances the effective inactivation of bacteria through the action of the metallic particles.

In addition to GO, CNTs and CQDs have also been utilized in the generation of antimicrobial composites through electrospinning. Apart from GO, CNTs and CQDs have also been utilized in the generation of antimicrobial composites through electrospinning. However, in the case of electrospun fibers containing CNTs, the antimicrobial effectiveness is generally not directly attributed to the carbonaceous nanomaterial itself. Several researchers used nanomaterial additives to improve the mechanical properties and the cell proliferation process, while the antimicrobial activity is provided by other active components. For instance, chitosan-modified PCL-CNTs electrospun fibers were reported as antibacterial materials, with the efficacy of bacteria inactivation attributed to the presence of the biopolymer on the surface of the fibers [94]. Similar findings were observed by Lui et al. [95], where PLA electrospun fibers containing multi-walled carbon nanotubes (MWCNTs) and chitosan showed antibacterial activity primarily due to increased chitosan content. In the same way, another recent example involved composite materials incorporating Fe_3_O_4_/SrO_2_ and functionalized MWCNTs demonstrated antibacterial performance against *E. coli* and *S. aureus* [96]. CQDs were used in combination with CS, silk fibroin, and α-tricalcium phosphate to develop a mat for wound healing applications [97]. N-doped CQDs synthesized via the hydrothermal method were added to a blend solution of the biopolymers in trifluoroacetic acid, along with α-tricalcium phosphate for electrospinning. The obtained fibers (diameter ca. 100–500 nm) were then crosslinked using glutaraldehyde to improve the hydrophilic/hydrophobic properties. Antibacterial assays against *E. coli* and *S. aureus* were conducted to determine the MIC in each case. The addition of α-tricalcium phosphate to CQDs resulted in a decrease in the MIC for both bacterial species. Furthermore, the presence of silk fibroin and particularly chitosan led to significantly lower MIC values (1.58 ± 0.4 mg mL^−1^ for *E. coli*, and 1.76 ± 0.12 mg mL^−1^ for *S. aureus*). The authors attributed this enhanced antimicrobial activity to the polycationic structure of CS, which electrostatically interacts with the negatively charged groups of the bacterial cell surface. This interaction provokes an alteration in cell permeability and physiological functions, leading to bacterial death.

In another study, PAN electrospun mats were developed by Sirelkhatim et al. for the inactivation of both non-pathogenic and pathogenic fungi [99]. The researchers compared the performance of PAN-based nanofibers (with an average diameter of approximately 500 nm) with PAN films and microfibers that underwent a carbonization procedure. The study involved evaluating the cell viability of *S. cerevisiae* SK1 and *C. albicans* after 18 h of contact with the carbonaceous materials. The results indicated that the nanofibrous mats were more effective in inhibiting fungal growth compared to the other materials, with a more pronounced effect observed in the case of the pathogenic strain (*C. albicans*). This outcome can be attributed to the unique fiber morphology, porosity, and surface composition of the nanofibrous mats, which induce different interactions and adhesion between the cells and the material. This finding suggests that the PAN electrospun nanofibrous mats hold promise for applications involving the inhibition of fungal growth, particularly in the case of multi-resistant pathogenic strains.

Based on the examples discussed in this section, it is evident that the antimicrobial activity of the carbonaceous materials incorporated onto electrospun mats alone is not as strong as that observed in nanofibrous materials containing metallic nanoparticles derivatives or EOs. It is also important to mention that the mechano-killing mechanism observed in bacteria (especially in single-walled carbon nanotubes (SWCNT) [100] is not present when the nanomaterial is incorporated inside the fiber structure. However, due to the well-known photothermal and photodynamic activity of the CNT, GO, or PAN, it is possible to develop materials for microbial inactivation by applying radiation, taking advantage of the other benefits provided by these types of materials in electrospun scaffolds (see Section 3.4).

### 3.4. Photoactivable Molecules and Nanomaterials

Other approaches involve the use of photosensitizers to achieve photodynamic inactivation (PDI) of several microorganisms. As mentioned in Section 2, PDI operates under same mechanisms of PDT where photosensitizers generate ROS to destroy essential components of biofilm matrices, both on the cell surface and inside microbial cells [101].

**Figure 9:**
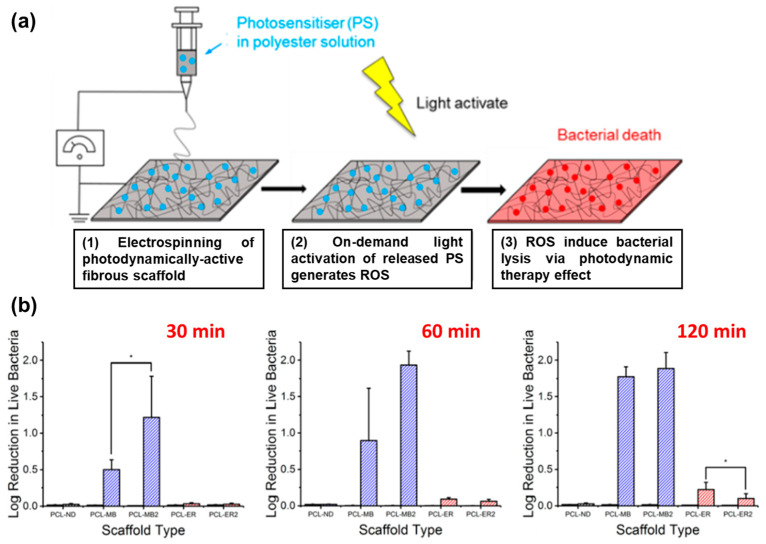
(**a**) Design and clinical applicability of photodynamically active electrospun fibers for antibiotic-free infection control. (**b**) Average log reduction in live bacteria cultured on PS-loaded PCL scaffolds following 30, 60, and 120 min of light exposure. Scaffolds PCL-MB2 and PCL-ER2 were electrospun from polymer solutions with doubled PS concentration ([PS] = 4.4 mM). Black bars refer to data obtained following incubation in the dark. Hashed bars represent light measurements. Results are reported as mean ± SD. The asterisk (*) denotes significantly different means (*p* < 0.05, *t*-test). Adapted with permission from reference *ACS Appl. Bio Mater.* **2019**, *2*, 4258–4270 ([102]). Copyright 2019. American Chemical Society.

For instance, Tronci and coworkers developed electrospun mats for their use in photodynamic processes (Figure 9a). Polyesters approved by Food and Drug Administration (FDA) such as PCL and PLGA were used to incorporate methylene blue (MB) and erythrosine B (ER) as photosensitizers (PS) with high loading efficiencies [102]. The polymers were dissolved together with the PS in hexafluoro-2-propanol before the electrospinning process. The fiber mats were tested regarding the antibacterial PDT against *E. coli* irradiating them with an LED light source for 30, 60, and 120 min. Results revealed the effectiveness of the therapy in the case of PCL-loaded fibrous materials showing log reductions in bacterial viability under light exposure: 0.5 (30 min), 0.9 (60 min), and 1.8 (120 min) as depicted in Figure 9b. Higher light doses generated more ROS, thereby increasing the therapeutic effect. However, when the concentration of MB was doubled, no further increase in bacterial death was observed after 60 min. Differences were also found in the effectiveness of PDT when ER was used as the photosensitizer, particularly at longer irradiation times (120 min). In another study, they reported similar semicrystalline fibers incorporating MB at lower concentrations, ranging between 0.2–2.2 mmol L^−1^ [103]. The inclusion of MB during the electrohydrodynamic process led to a decrease in the mean fiber diameter compared to pristine PLGA/PCL fibers. Successful in vitro release experiments of MB demonstrated attachment and proliferation of L929 cells over a 7-day culture period Antibacterial studies against *E. coli* and *S. mutans* compared the ZOI at dark incubation and under irradiation (UV light) at 30 and 60 min. Moreover, in the case of *E. coli*, the dosage of PS did not reveal any effect.

**Figure 10:**
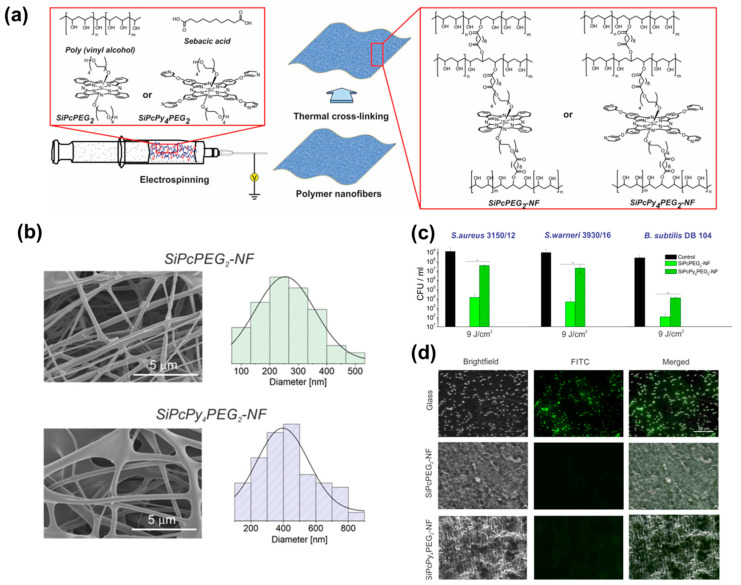
(**a**) Schematic representation of electrospinning of nanofibers and possible crosslinking process with premixed components. (**b**) SEM images and diameter distribution histograms of the PVA/SA-SiPcPEG_2_ and PVA/SA-SiPcPy_4_PEG_2_ nanofibrous mats. (**c**) Antibacterial effect of different nanofibrous mats after incubation with bacteria for 15 min and irradiation for 30 min. Error bars represent the standard deviation of three replicates, * *p* < 0.05 statistical difference versus control. (**d**) Representative fluorescence images of attached *B. subtilis* DB 104 cells (green) from a suspension of 10^10^ cells mL^−1^ after exposure to the glass cover slip, SiPcPEG_2_-NF, and SiPcPy_4_PEG_2_-NF for 1 h. Adapted with permission from *ACS Appl. Bio Mater.* 2020, *3*, 3751–3760 ([105]). Copyright 2020. American Chemical Society.

Phthalocyanines and their derivatives also present interesting photodynamic properties useful for the inactivation of pathogenic microorganisms. These compounds allow the incorporation of different functionalities and moieties due to their chemical versatility [104]. Strokov et al. employed a one-pot generation method for obtaining hydrophilic silicon(IV)phthalocyanine (SiPc)/PVA-based electrospun mats. Subsequently, a thermal cross-linking procedure based on esterification reaction with sebacic acid was performed (Figure 10a) [105]. After electrospinning, dense networks of non-woven fibers presenting diameters in the range of 250–400 nm were fabricated (Figure 10b). The concentrations of. SiPcPEG_2_-NF and SiPcPy_4_PEG_2_-NF were 13.28 nmol cm^−2^ and 24.92 nmol cm^−2^, respectively. The antibacterial efficacy of PS within the matrices was assayed against *S. aureus*, *S. warneri*, and *B. subtilis* (Gram-positive strains) as shown in Figure 10c,d. Irradiation was carried out using an LED lamp (λ = 660 nm) for 15 min (4.5 J cm^−2^) and 30 min (9 J cm^−2^). SiPcPEG_2_-NF achieved better results against *B. subtilis* (>5 log_10_ reduction) with 30 min of irradiation at 9 J cm^−2^. However, SiPcPy4PEG_2_-NF displayed lower antibacterial activity against the other strains (1.5 log_10_ for *S. aureus* and 1.5 log_10_ for *S. warneri*). Furthermore, the investigated materials exhibited excellent biocompatibility (assessed using dermal fibroblasts) and antifouling properties In another work, Sindelo and Nyokong incorporated ClIn(III) octacarboxy phthalocyanine (ClInOCP) and their conjugate with magnetic nanoparticles (MNP-ClInOCPc) into polyacrylonitrile (PAN) nanofibers (2 and 3.7 mg of each component) [106]. The photophysical studies of the materials showed singlet oxygen quantum yields (ΦΔ) of 0.36 (for the phthalocyanine conjugated with the magnetic particle-composed fibers) and 0.22 (for the fibers loaded with phthalocyanine). The inactivation of microbes in the water sample resulted in being higher for the magnetic conjugated phthalocyanine embedded into the PAN (ca. 63.7%) than fibers containing only ClInOCPc (<50.0%). Although the inactivation rate through the photodynamic effect was lower than that observed with the PS in solution, it can be considered a promising starting point, particularly for the development of materials suitable for use as patches.

Another photoactivable molecule, 5,10,15,20-tetrakis(1-methyl-4-pyridinio)porphyrin tetra(p-toluenesulfonate) (TMPyP), was incorporated onto polystyrene fibers surface [107]. First, an industrial electrospinning technology was employed for the fiber’s obtention. This technology involves the simultaneous formation of charged liquid jets on the surface of a thin wire electrode, allowing the fibers to be obtained with optimal positions for the jets Then, the pristine polystyrene membranes were derivatized by generating azo linkages through click reactions, and TMPyP was subsequently bound to the surface nanofibers. The functionalized materials displayed a slight increase in fiber diameter and improved their hydrophilicity. The photophysical properties of TMPyP were thoroughly investigated, and photo-antimicrobial experiments were conducted against *E. coli,* employing a 400 W solar daylight simulator equipped with a filter able to eliminate the heating effect. The assays led to the conclusion that the PDI process exhibited high efficiency, which was attributed to the effective photogeneration of antibacterial O_2_ (^1^Δg) from the TMPyP photosensitizer.

The photodynamic inactivation using carbon materials embedded into electrospun fibers has also been reported. Furthermore, PDI of PAN-based membranes containing carbonaceous materials was also reported in the last three years. CQDs synthesized by solvothermal methods were employed (ca. 0.6 and 2.5%) to electrospun together with PAN, obtaining fibers with a very uniform diameter size distribution (less than 500 nm) [108]. The assays were conducted by irradiating the samples with a xenon arc lamp (500 W) at a distance of 12 cm, equipped with a long-pass filter (λ ≥ 420 nm). Remarkably, illumination did not affect the mechanical properties of the mats. The photoactivated therapy exhibited moderate effectiveness against *S. aureus*. The authors achieved over 6 log units of inactivation against other strains such as *E. coli*, *B. subtilis*, and *P. aeruginosa*. The differences could be attributed to the singlet oxygen production that induces the PDI, being the *S. aureus* strain’s proneness to form clusters that limit the interaction between the CQDs and the bacteria. Pérez-Marquez and coworkers reported PAN electrospun mats containing graphene quantum dots (GQDs) with an optimized composition at 1% *w*/*w* of nanoadditive, processed using the electrohydrodynamic technique for 90 min [109]. The antibacterial test against *E. coli* was designed by incubation (37 °C, 100% humidity) and irradiation using a fluorescent lamp (18 W, 30 cm of distance) at different times and a dark control for comparison. Bacterial viability was determined by counting CFU after extracting samples at different time intervals and calculating the log value per cm^2^ of the contact surface. The results revealed time-dependent decreases in CFUs due to ROS-mediated PDI: 4.4 CFU cm^−2^ at 1 h, 3.8 CFU cm^−2^ at 6 h, and 2.6 CFU cm^−2^ at 15 h. Moreover, complete inactivation was achieved after 24 h. The performance of GQDs was also compared to that of classical PS (violet crystal and toluidine), positioning the developed material as a potential candidate to be used as an antibacterial surface and as a replacement of antibiotic-loaded patches.

Another novel material to combat fungi multi-resistance was reported by Liu et al. [110]. They developed recyclable and biodegradable PLA-hypocrellin A nanofibrous electrospun mats useful for the inactivation of C. auris, a microorganism classified into the critical priority group in the list of WHO fungal priority pathogens [111]. The reported material resulted in appropriate fiber diameter, mechanical properties, surface wettability, water vapor transmission rate as well as excellent biocompatibility, being interesting for its application as wound dressings. Regarding the antifungal activity, it was tested by irradiation with 470 nm (100 mW cm^−2^) laser source to evaluate the antimicrobial photodynamic inactivation. Several kinds of experiments were carried out to evaluate the in vitro and in vivo performance of the PLA-hypocrellin A nanofibers, including antifungal activity assays, fluorescence imaging, hemolysis test, extracellular and intracellular ROS and ^1^O_2_ detection, determination of DNA fragmentation, skin wound infection analysis in the Sprague Dawley rat model, and histopathologic assays, among others. As a summary of the performed studies, the authors suggested that the mechanism of inactivation is due to the materials mediated photodynamic effect having strong ability for ROS generation, which may provoke direct damage to cells since they induce and regulate apoptosis, followed by DNA fragmentation and morphological changes in nucleus cells (condensation).

Table 1 summarizes the electrospun materials containing antimicrobial agents described in the present section and the most relevant characteristics regarding the inhibition or inactivation of microorganisms’ strains.

## 4. Antimicrobial 3D Printed Materials

The design and fabrication of biomedical devices require a combination of biocompatible materials and a precise control over the three-dimensional structure to obtain non-porous or highly porous materials with interconnected pores, depending on the specific application. Moreover, it is crucial to ensure suitable surface properties [112], controlled hydrolytic stability [113], abiomechanical properties matching the native tissues [114,115,116], and, of course, biocompatibility [117,118] and biodegradation properties [119,120]. The use of medical devices has grown in the last few years, mainly due to the rise in life expectancy. However, this, coupled with the overuse and misuse of antibiotics, as well as the natural resistance of certain bacteria to different antibiotics, has led to the emergence of multi-resistant bacteria and fungi, posing a significant problem.

**Figure 11:**
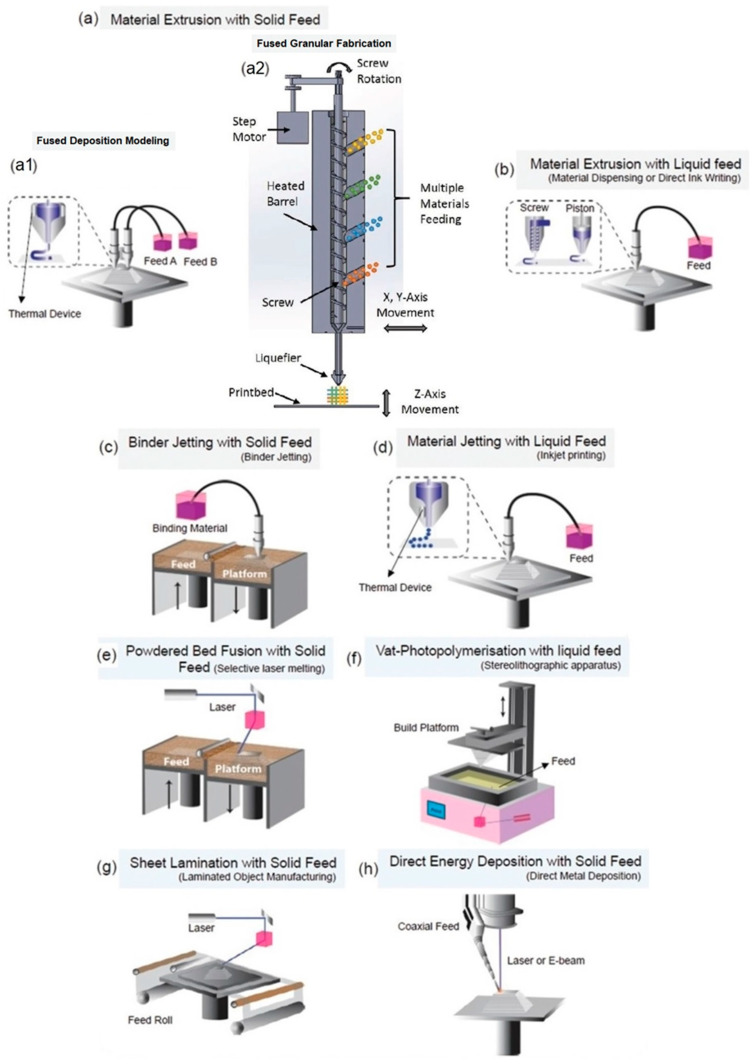
ASTM-classified AM processing techniques. (**a**,**b**) material extrusion: solid feed (**a1**) FDM and (**a2**) FGF, or liquid feedstock (**b**) DIW, (**c**) binder jetting, (**d**) material jetting, (**e**) powder bed fusion, (**f**) vat polymerization, (**g**) sheet lamination, and (**h**) direct energy deposition. Adapted with permission from *Adv. Mater.* **2020**, *32*, 2000556 ([123]) and *AIP Conf Proc* **2016**, *1769*, 190,004 ([124]). Copyright 2020, Wiley-VCH; Copyright 2016, AIP Publishing.

AM, also known as 3D printing, solid freeform fabrication (SFF) or rapid prototyping (RP), is a highly versatile fabrication technology that enables the construction of 3D solid objects through a layer-by-layer process using computer-aided design (CAD) models [121]. This technology is particularly suitable for biomedical applications compared to traditional processing approaches, as it allows to produce complex scaffolds with precise geometric control at both macro and micro-scales, utilizing simplified manufacturing procedures. Furthermore, AM enables customized production, from a wide variety of biomaterials. According to ISO/ASTM 52900:2015, AM processing techniques can be classified into seven categories [122]: material extrusion such as fused deposition modeling (FDM, also called fused filament fabrication—FFF), fused granular fabrication (FGF) and direct ink writing (DIW); binder jetting; material jetting; powder bed fusion processes such as selective laser sintering (SLS) and selective laser melting (SLM); vat photopolymerization such as stereolithography (SLA), digital light processing (DLP) and volumetric 3D printing; sheet lamination; and directed energy deposition (Figure 11).

Despite its huge potential, there are still relatively few studies that have explored the use of 3D printing structures with antibacterial properties. Recently, antibacterial 3D printing methods have mainly focused on preprint loading methods, such as surface coatings, preloaded filaments, and resins [125,126]. By far, the most employed techniques for this application have been FDM [127], FGF [128], DIW [129], SLA [130], and DLP [131]. In the FDM approach, a filament based on a thermoplastic material is fed, melted and extruded through a heated nozzle. The material quickly solidifies upon extrusion to form individual layers, eventually building the desired object. For complex shapes, a support structure may be necessary during the printing process, which can be removed once printing is complete. FGF, also called granular FDM or pellet AM, among other names, is a less-known, simpler and faster alternative to FDM. Instead of using filaments, this methodology employs granules or pellets that can be directly fed into an FGF printer [122,132,133]. As a result, plastic waste can be easily recycled after a straightforward pelletizing step, aligning with the principles of a circular economy. The FGF technique requires a distinct type of extruder in comparison to FDM. Two main strategies have been explored: plunger-based and screw-based FGF. The former relies on a heated syringe as a pellet reservoir, with the material extruded through the application of mechanical or pneumatic pressure [134]. The latter is the preferred strategy, as the screw-based approach allows for constant feeding. In this method, the granules are fed into a hopper, and a rotating screw inside a heated barrel extrudes the material through a heated nozzle to produce the final piece. Moreover, by eliminating the filament fabrication step, this approach reduces the number of heating processes, minimizing the potential thermal degradation of the thermoplastic material. Multi-material processing can also be achieved, facilitating the evaluation of composition-property relationships. DIW is a technique that utilizes composite or blend liquid or paste-like materials, known as inks, typically but not exclusively hydrogel-based materials. These inks are extruded from a nozzle, with or without heating, with an extrusion methodology similar to that described for FGF [135]. Inks are materials that can be engineered to exhibit appropriate rheological behavior. It is crucial to carefully control the rheological properties of the inks to ensure their ability to support their own weight and maintain shape fidelity after extrusion. The inks should exhibit high viscoelasticity both before and after extrusion, while also demonstrating good shear-thinning behavior during the printing process [122,136]. In other approaches, DIW can be assisted by UV or NIR for in situ photocuring, presenting the advantage of spatial and temporal control of polymerization and enhancing the ability to construct complex structures [137,138].

SLA involves the use of a vat containing a liquid photopolymer-based resin. When exposed to a laser source, the resin undergoes crosslinking to form thin layers of solid material, eventually building complex structures [139]. Crosslinking can be achieved through either top-down or bottom-up approaches. In the top-down approach, a scanning laser is positioned above the vat to cure a layer of resin, after which the platform is lowered into the vat to repeat the process for subsequent layers. In the bottom-up approach, the laser is positioned at the bottom of the vat, and after curing the first layer, the platform is raised to cure the next layer. This process is repeated until the final 3D structure is achieved. The DLP technique is a variation of SLA, but instead of using a laser beam, it employs a UV digital projector. The projector enables the whole layer to be exposed and cured simultaneously in a single step. Additionally, DLP uses a shallower vat of resin, resulting in a significantly faster process and reduced material consumption [140,141]. In vat polymerization printing, it is common practice to subject the printed constructs to post-printing light exposure. This step helps enhance the stability and mechanical properties of the printed objects, ensuring optimal performance.

In the following subsections, we will provide an overview of the recent advancements in AM specifically focused on the development of 3D scaffolds with antibacterial properties.

### 4.1. Natural Polymers

Recent studies have highlighted the use of natural polymer-based materials, particularly CS, in the development of antibacterial 3D printed structures. CS, derived from the deacetylation of chitin, which is the second most abundant polysaccharide on Earth, offers numerous advantages. These include biocompatibility, biodegradability, ease of processability, antioxidation, antibacterial properties, and the ability to promote the growth of hydroxyapatite (HA) when immersed in simulated body fluid (SBF) [142,143]. This biopolymer has been extensively employed as a bioactive filler or coating in composite materials, often in combination with synthetic polymers possessing good mechanical properties [144]. This strategy aims to enhance the hydrophilicity, cell adhesion, bioactivity, and antibacterial behavior of the resulting materials.

Many studies have focused on the treatment of infected bone defects, where biofilm formation hinders proper healing. Consequently, the development of 3D bioactive scaffolds with bacteriostatic and bactericidal properties is of paramount importance for bone repair. Notably, several investigations have sought to improve the properties of PLA through the incorporation of CS. Wang et al. reported the preparation of 3D printed PLA porous scaffolds via FDM followed by surface modification with CS [145]. The obtained structures had a height and diameter of 9.9 mm and pore size of 0.3 × 0.3 mm^2^. The methodology consisted of immersion of the scaffolds in a solution of CS in acetic acid at different times (5 to 90 s), followed by transferring them to a sodium hydroxide solution for 1 min. The latter solution is a CS non solvent, employed to neutralize the acidic medium and trap the polymer on the surface of the scaffold. The procedure allowed for the functionalization of the PLA surface without significantly altering the bulk properties of the scaffold. Scaffolds immersed in the CS solution for 15 s exhibited desirable properties, including hydrophilicity and minimal changes in compressive modulus. CS was entrapped with a maximum depth of 24 μm and the formation of a surface apatite layer was observed after immersion in SBF for 1 day. This rapid in vitro mineralization suggests that the scaffold has high potential for use in a biomimetic environment. Moreover, it resulted in being non-cytotoxic to human fibroblasts. Although bactericidal behavior was not specifically tested in this study, the scaffold holds promise for the treatment of infected bone defects. Future studies could focus on evaluating the release behavior of CS from the PLA surface scaffold, considering its physical immobilization.

Other works reported strategies for the incorporation of CS into the bulk structure of PLA. Wu et al. reported the design of CS composite structures by grafting it onto PLA or PLA-g-MA (maleic anhydride) with different mass ratios (5/95, 10/90, 15/85, and 20/80) [127]. The samples were mixed uniformly and then extruded into filaments, which were subsequently 3D printed by FDM technique. The addition of MA was aimed at improving the interfacial adhesion within the blend. Therefore, CS was more evenly distributed in the PLA-g-MA scaffold due to its similar hydrophilicity to MA. Both composite materials were found to be non-toxic to human fibroblasts. However, the MA grafted material showed superior mechanical properties and water resistance compared to the plain PLA composite. Moreover, as expected, CS enhanced the antibacterial activity of PLA and PLA-g-MA against *E. coli* and *S. aureus*. Mania et al. presented the co-extrusion of mixtures composed of PLA or a soft derivative (sPLA) with different ratios of CS (3 and 10% *w*/*w*), to achieve filaments composed of a physical mixture of the raw materials for printing 3D objects, such as biomedical tools or implants [146]. The increase in CS content led to filaments with a higher porosity, lower density, and thus lower mechanical properties, especially elongation at break and Young’s modulus. The presence of MA enhanced the overall performance of the scaffold, likely due to improved compatibility between the PLA and CS components. In addition, the average growth reduction in *S. aureus* and *E. coli* was dependent on CS content. Maximum average growth reduction was found for 10% *w*/*w* CS formulations, with 87% against *S. aureus* and 38% towards *E. coli*. Three-dimensional printed samples in the form of perforated cubes were prepared by FDM with maximum infill, in order to evaluate the feasibility of filament printing. Filaments containing 3% *w*/*w* CS resulted in less accurate objects with composition heterogeneity. However, these filaments exhibited a 15% higher compressive modulus, while filaments with 10% CS content were found to be non-printable.

**Figure 12:**
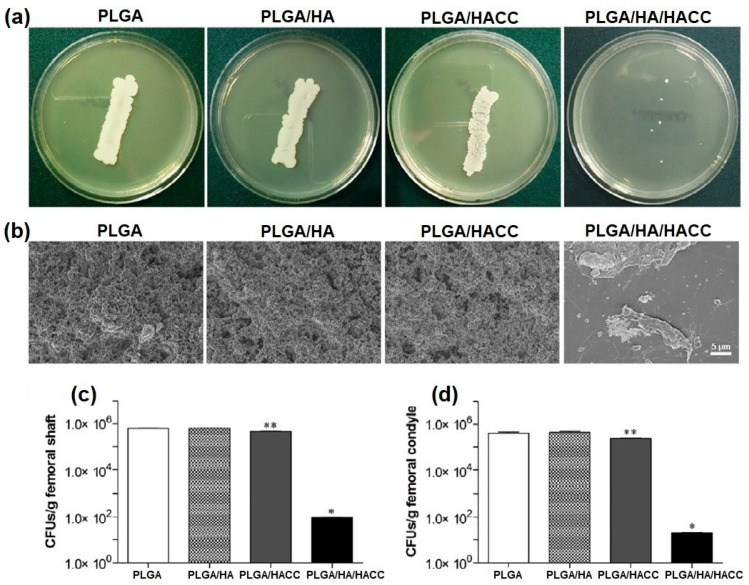
Microbiological assessment of internal implants and bone tissues. (**a**) Roll-over cultures of explanted stainless screws collected from rats. (**b**) SEM observation of biofilm formation on explanted polyethylene plates collected from rats. (**c**,**d**) Quantity of CFUs g^−1^ of pulverized femoral shaft and condyle collected from rats and rabbits, respectively. * *p* < 0.01 compared with the other scaffolds. ** *p* < 0.05 compared with the PLGA and PLGA/HA scaffolds. Reproduced with permission from *Acta Biomater.* **2018**, *79*, 265–275 ([129]) Copyright 2018, Elsevier Ltd.

PLGA was another polyester employed for the production of CS-based scaffolds. Peng et al. studied a chemical modification of CS to obtain a water-soluble quaternized derivative, hydroxypropyltrimethyl ammonium chloride CS (HACC) [147]. PLGA-g-HACC/HA 3D porous scaffolds were prepared from uniform pastes obtained by mixing PGLA particles with HA powder (particle size ~ 50 μm) in 1,4-dioxanone by heating under pressure using a 3D bioplotter. Then, HACC was covalently grafted to the 3D structure. These scaffolds led to a decrease in bacterial adhesion and biofilm formation under in vitro and in vivo conditions [148]. A further study with PLGA-g-HACC/HA similar scaffolds (PLGA:HA ratio 4:1) with dimensions of 6 × 4 mm^2^ and 24 layers were also 3D printed using a bioplotter. The study demonstrated that these bone substitutes significantly lowered the bacterial load and biofilm formation by methicillin sensitive *S. aureus* (MSSA) on their surface in contact with infected cortical and cancellous bone defects, expressed as CFU per gram of femoral chaft or condyle (Figure 12). This is a crucial behavior since the healing process is closely related to the prevention of film formation. Moreover, the scaffolds promote bone regeneration in in vivo animal models: female Sprague Dawley rats and skeletally mature female New Zealand white rabbits [129].

**Figure 13:**
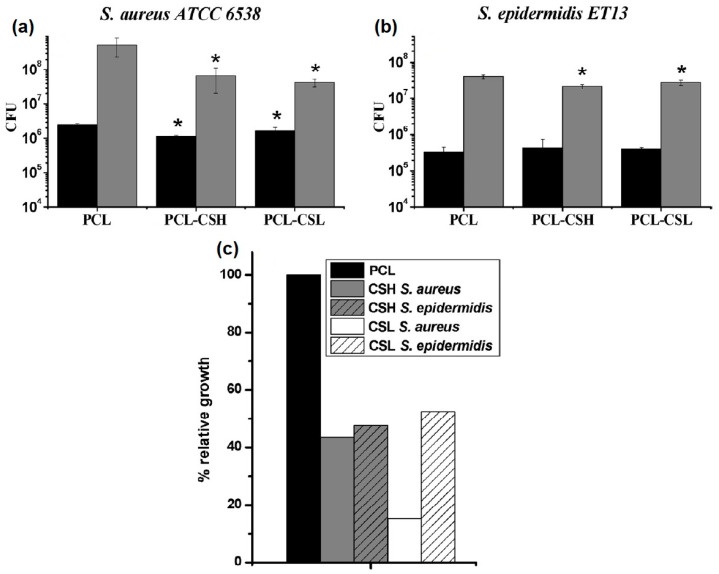
CFU recovered after 1 h adhesion (black) and after 24 h of biofilm formation (grey) with *S. aureus* ATCC 6538 (**a**) or *S. epidermidis* ET13 (**b**). Samples which are significantly different than the corresponding unmodified PCL scaffold under t-student test are marked with * (*p* < 0.05). (**c**) Relative bacterial growth after 24 h with respect to PCL (black columns) for CSH (grey columns) and CSL (plain columns) tested against *S. aureus* ATCC 6538 (plain columns) and *S. epidermidis* ET 13 (striped columns). Adapted with permission from *Carbohydr. Polym*. **2018**, *191*, 127–135 ([128]) Copyright 2018, Elsevier Ltd.

Surface functionalization of PCL scaffolds has also been performed. Scaffolds were prepared by a plotting process of melted pellets extruded under pressure, obtaining structures with a lay-down pattern of 0/90° and dimensions of 10 × 9 × 3 mm^3^ [128]. Then, CS with different molecular weights were employed as antibacterial agents by surface functionalization. First, methacrylic acid N-hydroxysuccinimide ester (NHSMA) was grafted onto the PCL surface after Ar-plasma/air activation. Finally, CS reacted with NHS groups for chemical bonding. All scaffolds displayed good L929 fibroblasts adhesion and viability. XPS results showed that high Mw CS (CSH) exhibited more efficient and more homogeneous coverage of the scaffold surface than low Mw CS (CSL). However, despite being neither bactericidal nor bacteriostatic (Figure 13a,b), CS-modified scaffolds displayed a clear reduction in *S. aureus* and *S. epidermidis* growth (Figure 13c). In fact, CSL modification displayed a more pronounced decrease in *S. aureus* growth rate than CSH. Future works regarding possible inflammatory reaction upon implantation could be explored.

ε-poly-L-lysine (EPL) is another natural polymer that has been explored for its incorporation into 3D printed structures through different approaches. EPL is a hydrophilic cationic polymer, water soluble, and the only polylysine naturally occurring in nature. Commercial EPL is obtained from fermentation of *Streptomyces albulus* and is mainly employed as a food preservative due to its inherent antibacterial and antifungal properties, non-toxicity, biodegradability, and low-cost production [149].

Tian et al. obtained PCL/HA (mass ratio 7:3, with HA particle size ranging from 1 to 20 μm) scaffolds by FDM [150], with dimensions of 2 × 2 × 2 mm^3^ and 10 × 10 × 5 mm^3^. The inclusion of HA particles resulted in structures with a slightly rough surface and an increase in the compression modulus compared to pure PCL. Scaffolds were then surface modified by immersion in an aqueous solution of EPL thrice. As expected, PCL/HA/EPL composite scaffolds exhibited enhanced hydrophilicity and water uptake, but a burst release of EPL was observed within the first hour. Scaffolds were evaluated in vitro with MC3T3-E1 osteoblast cell line. PCL/HA and PCL/HA/EPL proved to be cytocompatible, displaying cell attachment and proliferation. PCL/HA/EPL scaffolds displayed antibacterial in vitro activity against *S. aureus*, *E. coli*, and *S. mutans* for at least 3 days. Further studies should be performed to assess the potential of these scaffolds for infection prevention and tissue regeneration in vivo.

Other studies explored the use of DIW process to obtain antibacterial hydrogels containing EPL, mainly for wound dressing applications. Two works focused on the development of cellulose composite hydrogels. Fourmann et al. described the production of antibacterial nanocellulose (NC)-reinforced poly(N-isopropylacrylamide) (PNIPAM) hydrogels [138]. Inks were prepared from nanocellulose suspensions in NIPAM with ethyleneglycol dymethylacrylate (EGDMA) as photocrosslinker. EPL was added in the form of methacrylate (EPL-MA, 1 or 2.5% *w*/*w*) to confer antibacterial properties through UV-grafting. The 3D printing was carried out using plastic cartridges and extrusion through nozzles with compressed air. The system underwent post-printing polymerization using UV light in nitrogen atmosphere. Composites containing cellulose nanofibers presented a highly anisotropic microstructure and mechanical properties due to their alignment along the ink flow direction as a result of the shear and extensional forces in the print nozzle. Significant reduction in *S. aureus*, *S. arlettae*, *E. coli* and *P. fluorescens* growth was observed in both hydrogels containing EPL. This was a concentration dependent behavior, with hydrogels containing 1% and 25% EPL-MA showing approximately 60% and 80% growth reduction, respectively. The simplicity of the synthesis and processing methods, along with the ability to tailor their mechanical and antibacterial properties, highlights the potential use of these hydrogels in wound healing applications. Wang et al. developed novel printable inks from carboxymethyl cellulose (CMC) and EPL [151]. Firstly, CMC and EPL were separately reacted with glycidyl methacrylate (GMA) to obtain photocurable derivatives. Then, aqueous solutions with different CMC-GMA/EPL-GMA mass ratios were loaded into a syringe and extruded through a nozzle using compressed air. The printed samples were photocured to obtain different structures (cube: 1.5 cm × 1.5 cm × 2 mm; round shape: 1.5 cm diameter). By adjusting the mass ratio of CMC-GMA and EPL-GMA, the pore size, mechanical strength, swelling, and degradation behavior of the hydrogels could be customized. Hydrogels exhibiting a high compression modulus (238 kPa), remarkable inhibitory effects (95%) against both *E. coli* and *S. aureus*, and antioxidant ability were obtained. To assess the healing efficacy of these hydrogels, a rat full-thickness skin defect model infected with *S. aureus* was established. Hydrogels could significantly promote granulation tissue growth, collagen deposition, and revascularization processes, leading the wound healing process by five times compared to the control group. Moreover, the hydrogels could effectively inhibit bacterial growth and remove ROS generated at the wound site. This work provides a new strategy for fabricating multifunctional hydrogels with antibacterial and antioxidant properties, which can be customized using 3D printing technology. These hydrogels show promising potential as wound dressings for healing large and irregular-shaped skin wounds.

In a different approach, a synthetic EPL was employed. Murphy et al. synthesized asymmetric triblock copolypeptides, comprising EPL (P1) or polyglutamic acid (P2) as central block and polycysteine and polytyrosine as lateral blocks. These materials were employed to prepare polypeptide-based hydrogels [152]. EPL was incorporated into P1 to confer antimicrobial properties, while polyglutamic acid in P2 served as a negative control. Moreover, polycysteine and polytyrosine lateral blocks were employed to provide hydrogel stabilization. The former was selected for crosslinking through covalent bonding, while the latter produces hydrogen bonding and hydrophobic interactions through a π–π stacking. These hydrogels exhibited high water content and could be processed into complex 3D structures with excellent shape retention at concentrations ranging from 0.75% to 2% *w*/*w*. P1 hydrogel demonstrated antimicrobial activity in a contact-killing assay, resulting in a reduction of 7.6 CFU mL^−1^ for S. aureus and 6.5 CFU mL^−1^ for *E. coli* at a concentration of 1.5 wt%. In contrast, the control hydrogel P2 exhibited a negligible reduction in CFUs at 1.5 wt%. These hydrogels hold great potential as a versatile platform for designing scaffolds for a wide range of biomedical applications.

### 4.2. Metallic Nanoparticles and Derivatives

As mentioned before, the incorporation into polymeric systems of metallic and metallic-derived nanoparticles with antibacterial activity towards multi-resistant pathogens is promising for the design of matrices for medical applications such as wound dressings, among others. Different methodologies exploring this strategy have been reported in the last several years.

Some works explored the incorporation of copper and silver into polymeric matrices due to their high antimicrobial activity against wound infections caused by antibiotic resistant bacteria [153,154]. Two investigations focused on incorporating copper into hydrogels have been reported. The cytotoxicity of copper varies depending on the polymer chemistry, with hydrogels showing a higher effect due to the easier diffusion and release of copper ions [155]. Yang et al. obtained hydrogels containing copper ions by one-step electrophoretic deposition (EPD) as a novel and rapid 3D printing technique [156]. EPD is an attractive methodology that provides a facile and controllable strategy for depositing particles onto an electrode through an external electric field [157]. The technique enables the assembling of biopolymers with controllable properties at a low cost [158], making it well-suited for 3D printing applications [159]. Moldable hydrogels were prepared from citrus peel pectin (CPP) solutions (0.75% *w*/*w*), employing a copper plate as anode, and a voltage of 3 V for 15 min [156]. The oxidation of Cu led to CPP hydrogels loaded with Cu^2+^ which could easily coordinate with CPP carboxylate groups. The release of Cu^2+^ from the as-prepared systems provided antibacterial activity towards *E. coli* and *S. aureus* probably due to their catalytic behavior to produce ROS. One of the main challenges of this technique is to achieve complex structures, due to the intrinsic process, added to pH and temperature changes that may make difficult the production of homogeneous structures. Furthermore, other negatively charged macromolecules and metals could potentially be used for functional electrogel formation with antibacterial properties for wound healing and bone repair applications. Gutierrez et al. developed another novel methodology for the preparation of 3D printed structures from alginate (AG)-Cu nanoparticles [160]. AG crosslinking was performed with calcium ions, followed by an ion exchange with Cu^2+^. It was observed that direct crosslinking with Cu^2+^ led to higher AG degradation during the next step of the synthesis, and lower resolution of printed structures. Then, Cu ions were in situ reduced to obtain AG hydrogels incorporating Cu NPs. The presence of AG produced Cu NPs with lower diameter than the obtained from Cu^2+^ aqueous solutions. Inks were prepared by adding bacterial cellulose (BC) to improve printability of 3D scaffolds. Hydrogel structures of 30 × 30 × 1 mm^3^ (length × width × height) that were 3D printed with four layers and a mesh-like inner pattern with 1.5 mm of thread spacing were fabricated by DIW. The AG-based inks were printed at room temperature, and ionically crosslinked during extrusion by ionotropic process employing calcium solutions to produce scaffolds with long-term dimensional stability. Pure AG structures shrank during ionic crosslinking, whereas BC/AG scaffolds possessed long-term stability with no dimensional or volumetric changes. Moreover, scaffolds containing copper ions or nanoparticles displayed antimicrobial behavior against *E. coli* and *S. aureus*. Finally, similar scaffolds exhibited the ability to promote fibroblast proliferation without any inflammatory response, and favor in vivo angiogenesis [161]. This simple methodology enables the production of a new family of inks with high printability for potential applications in regenerative medicine and tissue engineering.

The main goal of antimicrobial materials is to possess a broad microbicidal spectrum and at the same time low toxicity to host tissues and cells. However, the functionality and the performance of an antibacterial biomaterial are by far more complex than choosing an antibacterial agent and a polymeric or composite matrix. As an example, silver is one of the most common antibacterial agents employed for wound healing, while some of its commercial products are more cytotoxic than microorganisms [162]. Therefore, when considering a potential formulation, i.e., as wound dressing, the nature of the dressing, its affinity for moisture, and the antibacterial agent and content must be carefully considered [163]. Even more, the biological evaluation of potential wound dressings can sometimes show a lack of correlation between in vitro and in vivo assays. In the case of silver-based wound dressings, this behavior could be due to the rapid inactivation of silver ions in physiological medium [164].

**Figure 14:**
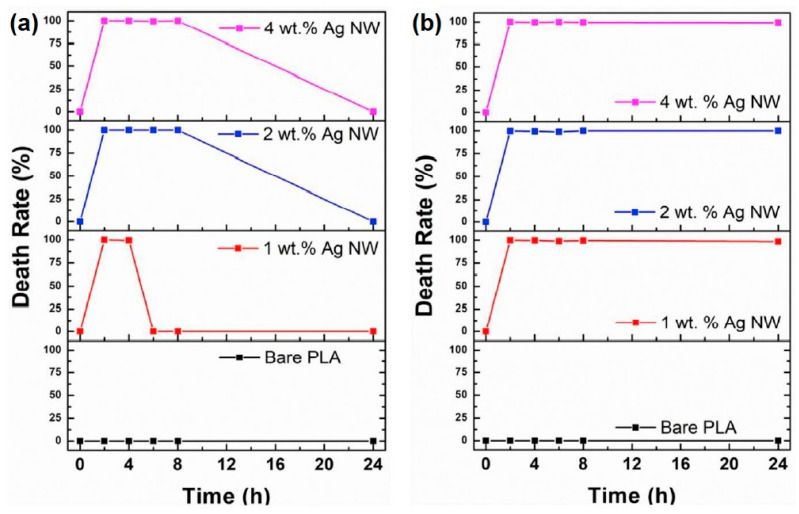
Death rate (%) graph for time kill assay carried out for 2, 4, 6, 8, and 24 h against (**a**) *S. aureus* and (**b**) *E. coli.* Adapted with permission from *Compos. B. Eng.* **2019**, *172*, 671–678 ([166]) Copyright 2019, Elsevier Ltd.

Several works explored the potential of silver-based structures as antibacterial and antifungal materials. Three reports evaluated the incorporation of silver into PLA scaffolds obtained by FDM. Yurttas et al. prepared filaments from PLA and wood flour (WF) containing Ag NPs [165]. The NPs were obtained in situ using a *Liquidambar orientalis* leaf extract as a reducing agent. The scaffolds exhibited antibacterial activity against *E. coli* and *S. aureus*, as well as antifungal activity against *C. albicans*. Bayraktar et al. obtained 3D printed structures from PLA and silver nanowires (Ag NWs) [166]. Briefly, Ag NWs were synthesized by the solution-based polyol method [167], dispersed and added to a PLA solution. The resulting dispersion was dried to obtain small Ag NWs-PLA granules that were extruded into filaments. Finally, non-porous disks with 5 mm diameter and 1 mm thickness were obtained by FDM with 0/90° printing orientation. Ag NWs resulted homogeneously dispersed within the PLA matrix. Disks were plasma etched in order to achieve direct contact between Ag NWs and bacteria strains. The 3D printed nanocomposites loaded with 4% *w*/*w* of Ag NWs were capable of killing 100% of *E. coli* and *S. aureus* bacteria in 2 h, and their antibacterial effect remained for 8 h against *S. aureus* and 24 h against *E. coli* (Figure 14). These materials are promising for the design of public area devices and personal products, but the Ag NWs concentration would probably be higher than that required for biomedical applications. Podstawczyk et al. reported a method for producing PLA-Ag NPs antibacterial filaments for FDM [168]. PLA and silver nitrate solutions were mixed to obtain solutions with different concentration (0.01 to 5% *w*/*w*). Solutions were then dried, and the resulting nanocomposites were extruded into filaments for 3D printing. Due to foaming during filament extrusion of 2.5% and 5% *w*/*w* formulations, these filaments were prepared by a solvent-free method. Silver nanoparticles (Ag NPs ≤ 20 nm) were produced in situ by thermal reduction during extrusion of the filaments. Cuboids of 10 × 10 × 5 mm^3^ were printed by FDM with high resolution and 95% infill for antimicrobial tests. The incorporation of Ag NPs into PLA did not significantly change its bulk properties. As expected, antibacterial activity against *S. aureus*, *E. coli* and *P. aeruginosa* was dependent on Ag NPs concentration. Systems prepared from silver nitrate (AgNO_3_) 1–5% *w*/*w* solutions exhibited the highest antimicrobial activity. These filaments could be employed for manufacturing of antimicrobial objects, but as mentioned before, a high silver concentration would probably prevent its use for the design of medical devices.

Afghah et al. reported the preparation of 3D printed composites from a synthesized copolymer based on polycaprolactone-block-poly(1,3-propylene succinate) (PCL/PPSu) impregnated with AgNO_3_ [169]. Mixtures were printed from a metal heated syringe connected to a pneumatic dispensing system. Samples with dimensions of 10 × 10 × 2 mm^3^ (15 layers), a gap of 350 μm among filaments, and layer thickness of 150 μm were printed. The obtained diameter for filaments was 270 μm, and structure dimensions as well as silver distribution resulted in being highly homogeneous. In addition, the block copolymer scaffold displayed a higher hydrolytic and enzymatic degradation behavior than PCL. The 3D printed structures containing AgNO_3_ concentrations below 5% displayed no cytotoxic behavior for human dermal fibroblast cells after a 21-day incubation period. Hence, the antibacterial activity of 5 × 5 mm^2^ polymer films with a 1 and 2.5% *w*/*w* AgNO_3_ content was tested against *P. aeruginosa* and *E. coli*, *S. aureus* and yeast *C. albicans*, which are the main bacteria responsible of infections related to implants or burn wound areas [170]. The samples inhibited *C. albicans* growth and, to a lesser extent, *P. aeruginosa*, *E. coli*, and *S. aureus* antimicrobial activities were observed. The low processing temperature of this technique, added to the high degradation rate of the evaluated system, could be suitable for the incorporation of temperature-sensitive bioactive agents. Therefore, these materials could be potentially considered for biomedical applications, i.e., skin regenerative therapy.

Ekonomou et al. reported on the fabrication of 3D printed structures from commercially available filaments of PLA and thermoplastic polyurethane (TPU) loaded with CuO or Ag NPs (< 2% w/w) [171]. Structures with dimensions of 30 × 10 × 1 mm3 were printed with a 100% infill. Specimens were manufactured in three different orientations (XY, XZ, and ZX) relative to the build platform. The specimen with the highest tensile strength was the obtained with the XY orientation, followed by ZX and XZ orientations. The composite structures exhibited a slightly stiffer response compared to the plain ones. All CuO and Ag NPs-loaded surfaces displayed a higher inhibitory effect against biofilms of *L. monocytogenes* and *S. aureus* compared to those of *E. coli* and *S. typhimurium*. Additionally, these structures exhibited higher hydrophobicity than those made from plain polymers. This approach provides new insights for the development of antimicrobial 3D printed surfaces and equipment, enabling their application in inhibiting the colonization of most common nosocomial and foodborne pathogens, and reducing the risk of cross-contamination and disease outbreaks.

Buj-Corral et al. employed metal–PLA composite filaments (copper-filled PLA, bronze-filled PLA, and steel-filled PLA) to produce scaffolds with enhanced mechanical properties compared to plain polymeric scaffolds [172]. Disk-shaped samples of 6 mm in diameter and 2 mm in height, with line spacing values of 0.6, 0.7, and 0.8 mm were manufactured. Among these, only steel-filled PLA scaffolds displayed cytocompatibility with human bone marrow-derived mesenchymal stromal cells and differentiation towards osteoblasts, suggesting potential applications for bone tissue engineering. However, the scaffolds were not assessed for their antibacterial activity.

**Figure 15:**
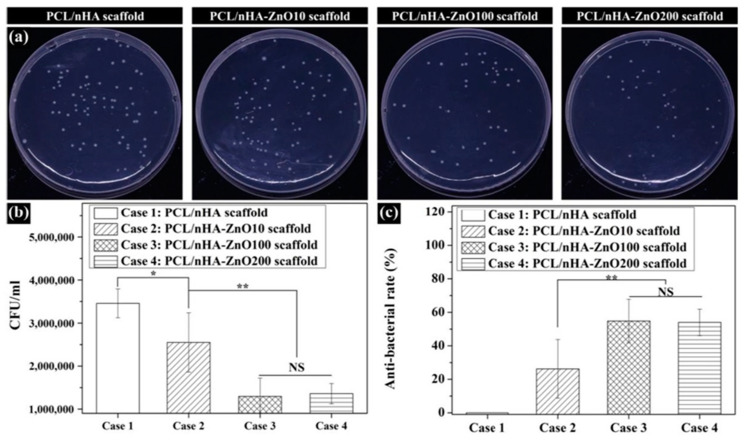
Assessment of antibacterial activity of fabricated scaffolds: (**a**) *E. coli* images recultured on agar for 24 h; (**b**) CFU; (**c**) antibacterial rate (NS: nonsignificant; * *p* < 0.05, ** *p* < 0.01). Reproduced with permission from *Polymers* **2020**, *12*, 2193 ([175]) Copyright 2020, MDPI.

The incorporation of zinc to scaffolds for tissue engineering has also been employed to prevent surgical site infection. Zinc ions present not only antibacterial activity [173], but also improve cell adhesion, promote differentiation of mesenchymal stem cells, and stimulate bone metabolism [174]. Cho et al. prepared 3D printed scaffolds from PCL/nHA (10% *w*/*w* nHA/PCL) with a kagome structure [175]. The kagome structure, also called supercell structure, is a combination of hexagons and triangles known to provide an excellent relative strength [176,177]. Structures with dimensions of 5 × 5 × 3.6 mm^3^, a porosity of 50%, and an apparent pore size of 500 μm were obtained. The composite raw material was extruded by a single screw under pressure with heating. Then, ZnO coatings of 10, 100 and 200 nm were performed by sputtering. The coated scaffolds exhibited a lower compressive modulus, attributed to the observed chain scission due to ZnO sputtering [178]. However, the compressive modulus was superior to other previously reported 3D printed scaffolds because of its HA reinforcement and its novel kagome structure [178]. Although in vitro cell viability was not affected by the presence of ZnO, 100 nm or higher thick coatings led to an increase in the in vitro cell proliferation of human osteosarcoma cell line and antibacterial activity against *E. coli* (Figure 15). Finally, considering its mechanical properties, in vitro cell response, and antibacterial activity, PLA/HA printed scaffolds with 100 nm-thick ZnO coatings could potentially be employed for bone regeneration and prevention of surgical site infection. Luo et al. also obtained 3D printed scaffolds from PLA incorporating ZnO NPs through surface modification of halloysite nanotubes (HNTs) [179]. Halloysite is an aluminosilicate clay naturally existing in the form of nanotubes. HNTs are cytocompatible and biocompatible [180], being able for surface modification, and with the potential to enhance osteogenic differentiation and mechanical properties of a scaffold [181]. ZnO NPs were deposited on the surface of HNTs (30% *w*/*w*), and composite filaments were prepared and processed by FDM to obtain 3D structures of 6 × 6 × 2 mm^3^, an average pore size of 600 nm and 60% overall porosity. Due to the hydrophobicity of the scaffolds, a three-layer coating was performed by immersion in fetal bovine serum (FBS), sodium hydroxide and again in FBS to facilitate cell adhesion. Unfortunately, the as-prepared scaffolds were not able to inhibit *S. aureus* growth, so an additional layer of gentamicin had to be incorporated, preserving the osteogenic behavior. These composite matrices may serve as candidates for the fabrication of customized bone implants.

Vat polymerization techniques were also explored. Marin et al. obtained 3D printed structures by stereolithography (SLA) of an acrylic resin. Samples were designed as hexagons with a thickness of 3 mm, a diagonal of 12 mm, a surface tilt angle of 45°, and a nominal resolution of 25 μm. Poly(methyl methacrylate) (PMMA) samples were then spray coated to obtain a layer of about 50 μm containing powders of different nitrides (β-Si_3_N_4_, Hf_3_N_4_, Zr_3_N_4_ and AlN) [130]. The best dispersion was achieved by using hafnium nitride, the ceramic with lower particle diameter (about 1 μm). All coated samples displayed antibacterial activity against *E. coli* and *S. epidermidis*, while hafnium nitride showed the best performance against the former, and aluminum nitride was more efficient against the latter. The antibacterial mechanism of silicon and aluminum nitrides can be explained by their progressive oxidation that leads to a subsequent release of ammonia. Moreover, hafnium and zirconium nitrides, being more stable due to the formation of oxide passive layers, possess antibacterial properties both in the nitride and oxide forms. These results showed the ability of nitride coatings to improve in vitro antibacterial properties of PMMA implants obtained by a 3D printing technique used in orthodontics. This strategy could be employed to design 3D printed composite bone screws.

Aati et al. developed 3D printed denture base resin materials incorporating mesoporous silica nanocarrier loaded with silver (Ag/MSN) at concentrations up to 2% *w*/*w* relative to the commercial resin (C&B, NextDent, Soesterberg, The Netherlands) in order to enhance mechanical and antifungal properties [182]. Specimens with different geometries were obtained by DLP. The addition of Ag/MSN significantly improved surface hardness and crack propagation resistance. However, flexural strength decreased when the Ag/MSN concentration exceeded 1 wt%. The specimens resulted in being non-cytotoxic toward fibroblasts, while the antifungal activity against *C. albicans* correlated with the concentration of Ag/MSN. The inclusion of Ag/MSN in the acrylic resin denture base material showed a significant enhancement in antimicrobial activity against *C. albicans*, which is the main cause of denture stomatitis. The antifungal activity persisted even after 3 months of aging in artificial saliva to simulate the oral physiological conditions. These researchers also explored the incorporation of zirconia (ZrO_2_) NPs into the commercial resin [183]. The ZrO_2_ NPs were functionalized with 3-methacryloxypropyltrimethoxysilane (γ-MPS) and mixed with the dental resin at concentrations up to 5% *w*/*w*. Structures with a cylindrical shape and dimension of 10 × 2 mm^2^ were obtained by DLP, post-cured, and placed in artificial saliva for 2 days or aged for 3 months. As for the previously reported materials, the specimens were non-cytotoxic to human oral fibroblasts, and the antimicrobial activity against *S. mutans* and *C. albicans* significantly improved with increasing filler concentration. Although there was a decrease in antimicrobial efficacy after aging, the modified resins still exhibited anti-biofilm properties. Therefore, the modification of the commercial resin with ZrO_2_ NPs or Ag/MSN holds promise in the dental field for fabricating long-term provisional restorations.

### 4.3. Essential Oils

As detailed in Section 3.2, the incorporation of EOs into scaffolds is a strategy employed for the preparation of antimicrobial scaffolds, and particularly antifungal scaffolds. A research group reported on the preparation of 3D matrices by DLP with antifungal activity for denture applications [131,184]. It is known that phytoncide oils possess antimicrobial, anti-inflammatory, antioxidative, and anti-tumoral properties [185]. Two types of phytoncide oil extracts were employed: phytoncide oil type A (PA) extracted from *Pinus densiflora* and phytoncide oil type B (PB) from *Chamaecyparis obtuse* [131]. The EOs were micro-encapsulated and mixed with a commercial denture base resin (NextDent Denture 3D+, Vertex Dental BV) at different concentrations: 2, 4, 6, and 8 wt% for PA microcapsules, and 5, 10, 15, 20, and 25 wt% for PB microcapsules. A dispersant was added with a concentration of 20 wt% relative to the mass of microcapsules. Discs with a diameter of 15mm and a height of 5 mm were fabricated by DLP technique. The manufacturing process was carried out with a build angle of 45° and a light source emitting at 385 nm. Subsequently, a post-polymerization process was conducted for 5 min. All samples exhibited non-cytotoxicity towards human gingival fibroblasts and displayed antifungal activity against *C. albicans*. Discs containing 6 wt% of PA and 15 wt% of PB microcapsules showed the highest antifungal activity, which persisted for 4 weeks in artificial saliva. Considering the sustained antifungal activity, these resins have the potential to be utilized as removable denture bases. In a further study, researchers designed structures using the commercial resin with 5 wt% of PA microcapsules and post-polymerization times (PPTs) of 5, 10, 20 and 30 min [184]. Discs with a 10-min PPT displayed a high degree of polymerization and the highest antifungal activity, while the flexural strength did not show any significant differences compared to unloaded discs. Although this denture base resin exhibited properties and accuracy within the clinical acceptance range, further in vitro and in vivo studies are required before conducting clinical trials.

### 4.4. Other Strategies

Silicones, or poly(dimethyl siloxanes) (PDMS), are another family of materials widely intended for biomedical applications. Although PDMS present a wide range of applications, such as contact lenses, long-term implants, catheters, and aesthetic implants, they have been scarcely processed by 3D printing. The low elastic modulus and high viscosity of silicone inks comprise a challenge for 3D printing processing. Freeform reversible embedding technique (FRE) has been employed to obtain helical and cylindrical tubes, using a hydrophilic gel to support PDMS prepolymer resins [186], and cuffs for pulse oximeters [187]. Recently, direct silicone printing of auricular prostheses with various grades of flexibility and hardness for better fitting with anatomy has been reported [188]. Moreover, the use of rapidly curing low viscosity inks from a mixture of silicones with different modulus produced objects without dripping during processing. The rapid UV curing avoided the need for supporting materials. Silicone 3D printing is in its early stage and presents a huge potential for the design of implants with improved properties for different applications. However, although biocompatible, silicone is highly hydrophobic. This promotes bacterial adhesion that could lead to severe infection [189]. Depending on the application, there are several antimicrobial strategies available, including passive and active chemical approaches. The former comprises the surface modification to increase their hydrophilicity to avoid bacterial adhesion and biofilm formation (i.e., poly(ethylene glycol) (PEG); plasma activation, which also allows the absorption of antibacterial agents) [190]. The latter involves the use of antimicrobial agents (i.e., silver, free-radical producing agents) [191].

Among different conventional antiseptics mentioned before, polymer-iodine complexes, known as iodophors, are also usually used in wound dressings to prevent infection [192]. For example, PVP iodine (PVP-I) solutions are highly effective against a broad spectrum of bacteria, including multi-resistant bacteria [193]. Moreover, PVA 3D printed structures obtained by FDM have also been explored for iodizing (PVA-I) [194]. As PVP and PVA are water soluble polymers, crosslinking must be performed after 3D printing in order to obtain 3D solid structures. Meshes that are 3D printed with the shape of circular disks (6.0 mm diameter and 0.8 mm thickness) and vascular Y-stents were obtained from PVA filaments by FDM. After crosslinking with vapor glutaraldehyde in acidic medium (PVA-X samples), these 3D printed meshes and stents showed smoother and more compact filament arrangements. Iodine loading was performed by sublimation (PVA-I and PVA-X-I samples), leading to radiopacity, and *S. aureus* and *E. coli* growth inhibition due to iodine release. Thus, these materials are promising for the design of a wide variety of antimicrobial and high-visibility devices.

Table 2 summarizes the 3D printed materials containing antimicrobial agents described in the present section and their most relevant characteristics.

## 5. Conclusions and Outlook

Over the last decade, a significant number of research works have been published focusing on the antimicrobial activity of several system types, including those derived from nanotechnology. However, a large portion of these studies still rely on the use of antibiotics or other related compounds responsible for AMR. In other cases, the development of metallic-derived nanoparticles with several functionalities and carbonaceous-based nanomaterials (CNTs, GO-based, QDTs, etc.) has been successfully achieved to enhance antimicrobial activities without relying on antibiotic drugs. In addition, the use of EOs and natural compounds has gained attention due to their diverse antimicrobial properties, which can be further enhanced through encapsulation or loading in nanomaterials. Despite these advancements, the incorporation of nanomaterials and bioactive compounds capable of controlling the growth of microorganisms, thereby replacing AMR-causing compounds, has not been extensively explored using electrohydrodynamic techniques or AM technologies. In this review, we summarize the existing reports on these systems, discussing their strengths and weaknesses, with a focus on the past five years.

Both electrospinning and 3D printing are advanced manufacturing techniques that can be utilized to address challenges related to AMR in polymeric-based materials. While they have different approaches and applications, they offer unique advantages in combating AMR. Electrospinning is particularly suitable for creating fibrous scaffolds with a high surface area-to-volume ratio, which is beneficial for antimicrobial applications. It can help overcome AMR through controlled release of antimicrobial agents, enhanced surface interactions, and improved mechanical properties. However, there is a limitation in the number of in vivo studies assessing the effectiveness of electrospun materials against antimicrobial resistance. On the other hand, 3D printing offers several advantages for combating AMR. The customization and the ability to create complex geometries allow for the design and fabrication of patient-specific implants or devices with antimicrobial properties. Multi-material printing enables the development of antimicrobial materials by combining polymers, antimicrobial agents, or other additives, thereby enhancing their effectiveness. The 3D printing method also facilitates localized drug delivery and enables rapid prototyping and production. However, there are some disadvantages to consider. The progress in this field is relatively recent, as evidenced by the lower number of research studies compared to polymeric materials generated via electrospinning. However, we anticipate that the inclusion of antimicrobial nanomaterials and compounds in 3D printed materials will see a significant increase soon. One crucial area of focus will be the design and development of inks for bioprinting, as this approach can overcome some of the limitations associated with electrospinning, such as the use of organic solvents that may be harmful and toxic. The emerging trend of “green electrospinning” or the utilization of molten polymers for scaffold generation are alternative approaches that can address these concerns.

While in vitro antimicrobial assays have demonstrated significant progress in inhibiting various strains of bacteria (both Gram-positive and Gram-negative) and other microorganisms such as fungi (i.e., *C. albicans*), in vivo assays have only been conducted in a limited number of publications. This is a crucial aspect considering the potential applications of these materials as patches or wound dressings to eliminate microorganisms and promote cell proliferation and tissue regeneration in infected areas. It is desirable for the most promising antimicrobial electrospun or 3D printed material composites to eventually undergo clinical trials to assess their effectiveness against AMR. However, it is currently too early for most of the reviewed cases to reach this stage of development.

Lastly, it is worth mentioning the emergence of novel nanomaterials or organic molecules, including MOFs, phthalocyanine derivatives, CQDs, GQDs, among others, as additives for the development of antimicrobial platforms that leverage photodynamic inactivation (PDI). This promising therapeutic approach has demonstrated high efficacy in inactivating various strains of microorganisms. However, a major limitation is the use of UV radiation to generate ROS responsible for microbial death. It is well-known that UV light can not only destroy pathogens but also harm healthy cells if the power source and conditions are not strictly controlled. In this regard, combining PDI with other photo-based therapies, such as antibacterial photothermal therapy using near infrared radiation (NIR), presents an interesting challenge for the scientific community in materials engineering and pharmaceuticals. Applying these approaches using electrospun or 3D printed materials, as discussed previously, for the development of antimicrobial dressings or surfaces, could potentially mitigate the limitations associated with UV radiation and provide a more controlled and targeted treatment.

From our point of view, we believe that the field of antimicrobial scaffolds has enormous potential due to the wide range of possibilities and combinations between the techniques as well as possibilities of making post-processing modifications to improve the performance and effectiveness of the antimicrobial therapies, but also other physicochemical, surface, and biological features for their practical use helping to overcome the AMR.

## Figures and Tables

**Table 1:** Electrospun mats containing antimicrobial agents capable of inhibiting or inactivating different strains to avoid AMR.

Material Denomination	Polymeric Matrix Composition	Type of Antimicrobial Agent	Antimicrobial Agents	In Vitro Assayed Strains	In Vivo Model	Reference
GEL/PDA- Metal	GEL/PDA	Metallic nanoparticles and derivatives	Metal ions: Ag^+^, Mg^2+^, Zn^2+^, and Ca^2+^	*VRE* *B. subtilis*	-	[68]
PCL-ionic Ag	PCL	Metallic nanoparticles and derivatives	Ag+	*E. coli* *S. aureus*	Balb/c mice	[69]
PAA/PAH-ZnO NPs	PAA/PAH	Metallic nanoparticles and derivatives	ZnO NPs	*E. coli* *S. aureus*	-	[70]
PVA/PVP-ZnO NPs	PVA/PVP	Metallic nanoparticles and derivatives	ZnO NPs	*S. aureus* *E. coli* *K. pneumonia*, *P. aeruginosa*	-	[71]
Polyethersulfone-ZnO nanorods	Polyethersulfone	Metallic nanoparticles and derivatives	ZnO nanorods	*S. aureus* *E. coli* *S. epidermidis*	-	[72]
PVA-CuO NPs/MC extract	PVA	Metallic nanoparticles and derivatives	CuO NPs	*B. subtilis* *E. coli*	-	[73]
PLA-Ag2[HBTC][im])	PLA	Metallic nanoparticles and derivatives	(Ag2[HBTC][im])	*E. coli* *P. aeruginosa* *S. aureus* *M. smegmatiss*	Kunming female mice	[76]
CS/PVA-Cu^2+^ (HKUST-1)	Ch/PVA	Metallic nanoparticles and derivatives	Cu^2+^	*E. coli* *S. aureus*	Balb/c mice	[77]
PCL-PEP	PCL	EOs	PEP	*E. coli* *S. aureus*	-	[79]
PCL-CAR/THY	PCL	EOs	CAR THY	*S. Aureus* *E. coli*	-	[80]
PCL-THY	PCL	EOs	THY	*S. aureus*	Male SKH1 mice	[81]
PCL/GEL-CLV	PCL/GEL	EOs	CLV	*S. Aureus* *E coli*	-	[82]
PCL/GEL-PEs	PCL/GEL	EOs	PEs	*S. Aureus* *E. coli*	-	[83]
PCL-Nanogels (contanining CSEO)	PCL	EOs	CSEO	*P. aeruginosa* *E. coli* *K. pneumonia* *S. aureus*	-	[84]
CA-Rosemary/Oregano	CA	EOs	Rosemary Oregano	*E. coli* *S aureus* *C. albicans*	-	[85]
Gellan/PVA- Cinnamaldehyde	Gellan/PVA	EOs	Cinnamaldehyde	*C. glabatra* *C. albicans* *P. aeruginosa* *S. aureus*	-	[86]
HA/PVA/PEO- Zn NPs/Cinnamon	HA/PVA/PEO	EOs	Zn NPs/Cinnamon EO	*S aureus*	Wistar albino rats	[90]
EC/GT-Honey	EC/GT	EOs	Honey	*E. coli* *S. aureus*		[91]
CS/PVA-GO	PVA/CS	Carbon-based nanomaterials	GO	*B. cereus* *S. aureus* *S. spp* *E. coli*	Adult Wistar rats	[92]
CS/SF-CQDs	CS/SF	Carbon-based nanomaterials	CQDs	*E. coli* *S. aureus*	-	[97]
PLGA/CS-GOAg	PLGA/CS	Carbon-based nanomaterials	GOAg	*S. aureus* *E. coli* *P. aeruginosa*	-	[98]
PAN	PAN	Carbon-based nanomaterials	Carbonizated PAN	*C. albicans*	-	[99]
PCL/PLGA-MB PCL/PLGA-ER	PCL/PLGA	Photoactivable molecules/nanomaterials	MB ER	*E. coli*	-	[102]
PCL/PLGA-MB	PCL/PLGA	Photoactivable molecules/nanomaterials	MB	*E. coli* *S. mutans*	-	[103]
PVA-SiPC	PVA	Photoactivable molecules/nanomaterials	SiPC and derivatives	*S. aureus* *S. warneri* *B. subtilis*	-	[105]
PAN-ClInOCP	PAN	Photoactivable molecules/nanomaterials	ClInOCP	*S. aureus*	-	[106]
Polystyrene- TMPyP	Polystyrene	Photoactivable molecules/nanomaterials	TMPyP	*E. coli*	-	[107]
PAN-CQDs	PAN	Photoactivable molecules/nanomaterials	CQDs	*E. coli* *B subtilis* *P. aeruginosa*	-	[108]
PAN-GQDs	PAN	Photoactivable molecules/nanomaterials	GQDs	*E. coli*	-	[109]
PLA-Hypocrellin A	PLA	Photoactivable molecules/nanomaterials	Hypocrellin A	*C. auris*	Sprague Dawley	[110]

**Table 2:** 3D printed structures containing antimicrobial agents.

Material Denomination	Polymeric Matrix Composition	Antimicrobial Agents	3D Printing Technique	In Vitro Assayed Strains	In Vivo Model	Reference
PLA-CS PLA-g-MA-CS	PLA PLA-g-MA	CS	FDM	*S. aureus* *E. coli*	-	[127]
PLA-CS	PLA	CS	FDM	*E. coli* *S. aureus*	-	[146]
PLGA-g-HACC/HA	PLGA/HA	HACC	DIW	*S. aureus* *S. epidermidis* *MRSA*	Female Sprague Dawley rats/Female New Zealand white rabbits	[129,148]
PCL-CS	PCL	CS	FGF	*S. aureus* *S. epidermidis*	-	[128]
PCL/HA-EPL	PCL/HA	EPL	FDM	*S. aureus* *E. coli* *S. mutans*	-	[150]
PNIPAM/NC-EPLMA	PNIPAM/NC	EPL	DIW	*S. aureus* *E. coli* *S. arlettae* *P. fluorescens*	-	[138]
CMC-GMA/EPL-GMA	CMC-GMA	EPL	DIW	*E. coli* *S. aureus*	Sprague Dawley rats	[151]
Tyr-Lys-Cys copolypeptide	copolypeptide	EPL	DIW	*E. coli* *S. aureus*	-	[152]
CPP-Cu	CPP	Cu	Electrophoretic deposition	*E. coli* *S. aureus*	-	[156]
AG/BC/Cu	AG/BC	Cu NPs	DIW	*E. coli* *S. aureus*	-	[160]
PLA/WF-Ag NPs	PLA/WF	Ag NPs	FDM	*E. coli* *S. aureus* *C. albicans*	-	[165]
PLA-Ag NWs	PLA	Ag NWs	FDM	*E. coli* *S. aureus*	-	[166]
PLA-Ag NPs	PLA	Ag NPs	FDM	*E. coli* *S. aureus* *P. aeruginosa*	-	[168]
PCL/PPSu-Ag	PCL/PPSu	Ag	FGF	*P. aeruginosa E. coli* *S. aureus* *C. albicans*	-	[169]
PLA-CuO NPs PLA-Ag NPs TPU-CuO NPs TPU-Ag NPs	PLA PLA TPU TPU	CuO NPs Ag NPs CuO NPs Ag NPs	FDM	*L. monocytogenes* *E. coli* *S. aureus* *S. typhimurium*	-	[171]
PCL/nHA-ZnO	PCL/nHA	ZnO	FGF	*E. coli*	-	[175]
PMMA-(β-Si_3_N_4_, Hf_3_N_4_, Zr_3_N_4_, AlN)	PMMA	β-Si_3_N_4_, Hf_3_N_4_, Zr_3_N_4_, AlN	SLA	*E. coli* *S. epidermidis*	-	[130]
Resin-Ag/MSN	Resin	Ag/MSN	DLP	*C. albicans*	-	[182]
Resin-ZrO_2_ NPs	Resin	ZrO_2_ NPs	DLP	*S. mutans* *C. albicans*	-	[183]
Resin-PA Resin-PB	Resin	Phytoncide oil	DLP	*C. albicans*	-	[131]
Resin-PA	Resin	Phytoncide oil	DLP	*C. albicans*	-	[184]
PVA-I	PVA	Iodine	FDM	*S. aureus* *E. coli*	-	[194]

## Data Availability

No new data were created or analyzed in this study. Data sharing is not applicable to this article.

## Abbreviations

ΦΔ	Singlet oxygen quantum yield
β-Si_3_N_4_	Silicon nitride
γ-MPS	3-methacryloxypropyltrimethoxysilane
Ag NPs	Silver nanoparticles
Ag NWs	Silver nanowires
AG	Alginate
AlN	aluminum nitride
AM	Additive manufacturing
AMR	Antimicrobial resistance
ASTM	American Society for Testing and Materials
BS	Bacterial cellulose
CA	Cellulose acetate
CAD	Computer-aided design
CAR	Carvacrol
CBD	Chemical bath deposition
CEO	Cinnamon essential oil
CFU	Colony-Forming Unit
CG-MS	Gas Chromatography-Mass Spectrometry
ClInOCP	ClIn(III) octacarboxy phthalocyanine
CLV	Clove essential oil
CLXD	Chlorhexidine
CMC	carboxymethyl cellulose
CMC-GMA	carboxymethyl cellulose-glycidyl methacrylate
CNTs	Carbon nanotubes
CPP	Citrus peel pectin
CQDS	Carbon quantum dots
CS	Chitosan
CSEO	*Citrus sinencis* essential oil
Cu NPs	Copper nanoparticles
CuO NPs	Copper oxide (II) nanoparticles
DA	Dopamine
DIW	Direct ink writing
DLP	digital light processing
EC	Ethylcellulose
ECM	Extracellular matrix
EDC	1-Ethyl-3-(3Dimethyl aminopropyl) carbodiimide
EDS	Energy-dispersive X-ray spectroscopy
EE	Encapsulation efficiency
EGDMA	ethyleneglycol dymethylacrylate
EOs	Essential oils
EPD	Electrophoretic deposition
EPL	ε-poly-L-lysine
EPL-MA	ε-poly-L-lysine-methacrylate
ER	Erythrosine B
FAO	Food and Agriculture Organization of the United Nations
FDA	Food and Drug Administration
FBS	Fetal bovine serum
FDA	Food and Drug Administration
FDM	Fused deposition modeling
FE-SEM	Field Emission Scanning Electron Microscopy
FFF	Fused filament fabrication
FGF	Fused granular fabrication
FRE	Freeform reversible embedding
FTIR	Fourier-Transform Infrared Spectroscopy
GC	Gas Chromatography
GEL	Gelatin
GEL/PDA	Gelatin/polydopamine
GMA	glycidyl methacrylate
GO	Graphene oxide
GQDs	Graphene quantum dots
GT	Gum tragacanth
GTMAC	Glycidyl trimethylammonium chloride
HA	Hydroxyapatite
HAc	Hyaluronic acid
HACC	Hydroxypropyltrimethyl ammonium chloride chitosan
HKUST-1	Copper-based metal-organic framework
HNTs	Halloysite nanotubes
H&E	Hematoxylin and Eosin
ISO	International Organization for Standarization
MA	Maleic anhydride
MB	Methylene Blue
MBC	Minimum bactericidal concentration
MC	*Momordica charantia*
MIC	Minimum inhibitory concentration
MNP	Magnetic nanoparticles
MOF	Metallic organic frameworks
MRSA	Methicillin-resistant *Staphylococcus aureus*
MSN	mesoporous silica nanocarrier
Mw	Weight average molecular weight
MWCNT	Multi-walled carbon nanotubes
NC	nanocellulose
NF	Nanofibers
Nha	Nanohydroxyapatyte
NHDF	Normal human dermal fibroblast
NHS	N-hydroxysuccinimide
NHSMA	Methacrylic acid N-hydroxysuccinimide ester
NIPAM	N-isopropylacrylamide
NIR	Near Infrarred Radiation
NPs	Nanoparticles
OIE	World Organization for Animal Health
P1	asymmetric triblock copolypeptide comprising EPL as central block, and polycysteine and polytyrosine as lateral blocks
P2	asymmetric triblock copolypeptide comprising polyglutamic acid as central block, and polycysteine and polytyrosine as lateral blocks
PA	phytoncide oil type A extracted from *Pinus densiflora*
PAA	Poly(acrylic acid)
PACT	Photodynamic antimicrobial chemotherapy
PAH	Polyallylamine hydrochloride
PAN	Polyacrylonitrile
PB	phytoncide oil type B extracted from *Chamaecyparis obtuse*
PBS	Phosphate-buffered saline
PCL	Poly(ε-caprolactone)
PDA	Polydopamine
PDI	Photodynamic inactivation
PDMS	Poly(dimethyl siloxane)
PDT	Photodynamic Therapy
PEG	Poly(ethylene glycol)
PEO	Poly(ethylene oxide)
PEP	Peppermint essential oil
PEs	*Pinus radiata* bark extracts
PGA	Poly(glycolic acid)
PLA	Poly(lactic acid)
PLGA	Poly[(rac-lactide)-co-glycolide]
PMMA	Poly(methyl methacrylate)
PNIPAM	poly(N-isopropylacrylamide)
PPSu	Poly(1,3-propylene succinate)
PPTs	post-polymerization times
PS	Photosensitizer
PVA	Poly(vinyl alcohol)
PVP	Poly(vinylpyrrolidone)
ROS	Reactive Oxygen Species
RP	Rapid prototyping
SAXS	Small Angle X-ray Scattering
SBF	Simulated body fluid
SEM-EDS	Scanning Electron Microscopy/Energy-Dispersive X-ray Spectroscopy
SFF	Solid freeform fabrication
SLA	Stereolithography
SLM	selective laser melting
SLS	Selective laser sintering
SPAN	Sorbitan ester
SWCNT	Single-Walled Carbon Nanotubes
TEM	Transmission Electron Microscopy
THY	Thymol
TMPyP	Tetrakis(1-methyl-4-pyridinio) porphyrin tetra(p-toluenesulfonate)
TPU	thermoplastic polyurethane
UV	Ultraviolet visible
VRE	Vancomycin-Resistant *Enterococci*
WF	wood flour
WHO	World Health Organization
XPS	X-ray Photoelectron Spectroscopy
XRD	X-ray Diffraction
ZnO	Zinc oxide
ZnO NPs	ZnO nanoparticles
ZOI	Inhibition zones
ZrO_2_ NPs	zirconia nanoparticles
